# Vascular Smooth Muscle Cell-Specific BCAT2 Deficiency Attenuates Diabetic Atherosclerotic Calcification via Histone Propionylation

**DOI:** 10.34133/research.1052

**Published:** 2026-01-06

**Authors:** Lili Zhang, Yujie Yang, Wei Yuan, Zhiyin Dai, Lihua Li, Zhen Sun, Chen Shao, Guangyao Zang, Yongjiang Qian, Zhongqun Wang

**Affiliations:** ^1^ Department of Cardiology, Affiliated Hospital of Jiangsu University, Zhenjiang 212001, China.; ^2^ Institute of Cardiovascular Diseases, Jiangsu University, Zhenjiang 212001, China.; ^3^ Department of Pathology, Affiliated Hospital of Jiangsu University, Zhenjiang 212001, China.; ^4^ Department of Cardiology, the First Affiliated Hospital of Anhui Medical University, Anhui 230022, China.

## Abstract

**Background:** Vascular calcification is a major cause of adverse outcomes of acute cardiovascular events in diabetic patients. However, the effective therapeutic target for diabetic atherosclerotic calcification remains unclear. Branched-chain amino acid transaminase 2 (BCAT2), a key rate-limiting enzyme of branched-chain amino acid (BCAA) catabolism, may play a potential role in the development of diabetic complications. This study aimed to elucidate the role of BCAT2 in diabetic atherosclerotic calcification. **Methods:** Airflow-assisted desorption electrospray ionization mass spectrometry imaging (AFADESI-MSI) was employed to investigate the spatial distribution of metabolites in frozen arterial sections obtained from diabetic foot amputations. Single-cell RNA sequencing datasets from arteries of diabetic foot amputations were used to identify the expression of metabolic enzymes in the BCAA catabolism. ApoE knockout mice with specific deletion of BCAT2 in vascular smooth muscle cells (VSMCs) were generated, and a diabetic atherosclerotic calcification model was established to evaluate the impact of BCAT2 in diabetic atherosclerotic calcification. Further, the gene regulatory mechanisms of BCAT2 in diabetic atherosclerotic calcification were investigated. **Results:** BCAA catabolism was enhanced in the calcified anterior tibial arteries from diabetic foot amputation revealed by spatial metabolomics. Furthermore, BCAT2 was found to be up-regulated in VSMCs of calcified anterior tibial arteries from diabetic foot amputation by single-cell transcriptomics. Notably, VSMC-specific BCAT2 deficiency attenuated diabetic atherosclerotic calcification without sex bias. Further experiments revealed that branched-chain α-ketoacids (BCKA) supplement, especially α-keto-β-methylvaleric acid (KMV) and α-ketoisovaleric acid (KIV), promoted osteogenic differentiation of VSMCs and diabetic atherosclerotic calcification. Mechanistically, VSMC-specific BCAT2 deficiency suppressed the generation of BCKA-derived propionyl-CoA, mitigating histone propionylation at the promoter of RUNX2, and thereby osteogenic differentiation of VSMCs and diabetic atherosclerotic calcification. **Conclusions:** Our study demonstrates a previously unrecognized role of BCAA catabolism in diabetic atherosclerotic calcification and further delineates that the BCAT2–BCKA axis contributes to the osteoblastic differentiation of VSMCs by epigenetically modulating RUNX2 expression via histone propionylation.

## Introduction

Diabetic macrovascular complications are the main cause of death and disability in diabetes patients, of which vascular calcification (VC) is one of the key pathological mechanisms [1,2]. Calcification in atherosclerotic plaque can cause stiffness and decreased compliance of the vascular wall, and can induce atherosclerotic plaque rupture, which increases the risk of acute cardiovascular events [3]. Compared with nondiabetic patients, the atherosclerotic plaques in coronary artery of diabetic patients have larger necrotic core and more extensive calcification [4]. VC is an active process involving osteoblastic differentiation and mineralization of vascular smooth muscle cells (VSMCs) [5,6]. However, the molecular mechanisms underlying VC in diabetic atherosclerotic plaques have not been fully elucidated, and no effective interventions have been identified.

Branched-chain amino acid transaminase 2 (BCAT2) is the determinant enzyme that catalyzes the degradation of the branched-chain amino acid (BCAA) to catabolites branched-chain α-ketoacids (BCKA) [7]. BCAA metabolism has been shown to be involved in multiple cardiovascular diseases including myocardial infarction, ischemia–reperfusion injury, atherosclerosis, hypertension, and heart failure [8–10]. Furthermore, BCAA can inhibit liver glucose synthesis and thereby reduce blood glucose concentration [11]. BCAT2-catalyzed production of BCKA can promote a phenotypic switch to the synthetic phenotype in pulmonary artery smooth muscle cells (PASMCs) of contractile phenotype [12]. Then, the role and specific mechanism of BCAT2 in regulating VSMC osteogenic differentiation and diabetic atherosclerotic calcification remains to be further explored.

BCAA, containing valine, isoleucine, and leucine, are converted to BCKAs [α-keto-β-methylvaleric acid (KMV), α-ketoisovaleric acid (KIV), and α-ketoisocaproic acid (KIC)] by BCAT, followed by decomposition into the final products propionyl-coenzyme A (prop-CoA) and acetyl-CoA by branched-chain α-keto acid dehydrogenase (BCKDH) [13]. Acetyl-CoA derived from BCAA-BCKA ultimately enters the tricarboxylic acid cycle to participate in energy metabolism, or serves as a source of acyl groups to participate in posttranslational modification and regulate protein expression [14]. Acetyl-CoA derived from BCKA regulates the acetylation of PRDM16, which disrupts the interaction between PRDM16 and PPARγ, consequently inhibiting the browning of inguinal white adipose tissue and the process of thermogenesis [15]. Inhibition of BCAT2 leads to a decrease in acetyl-CoA, reduces P300-dependent histone acetylation, and thus suppresses the transcription of FASN and ACLY [16]. In arterial thrombosis, the valine metabolite KIV and prop-CoA have the strongest promoting effect on platelet activation. Mechanistically, prop-CoA enhances integrin αIIbβ3-mediated cell signaling by inducing propionylation modification of proteins (especially at the K255 site of TMOD3) [17]. Histone lysine propionylation is a protein translational modification that involves the addition of a 3-carbon propionyl group from prop-CoA to the ε-amino group of a lysine side chain to regulate gene expression [18,19]. Runt-related transcription factor 2 (RUNX2), as a core transcription factor, dominates osteogenic transdifferentiation of VSMCs and atherosclerotic calcification [20]. The expression of RUNX2 is elevated in atherosclerotic calcified vascular tissue specimens, while it is minimally expressed in normal vascular tissue specimens [21,22]. However, the related regulatory mechanisms of BCAT2-mediated BCAA catabolism in regulating RUNX2 expression and VSMC osteogenic differentiation are still unclear.

In this study, we found that BCAT2, an outstanding metabolic enzyme in the BCAA catabolism pathway, was highly expressed in calcified arteries from diabetic foot amputation. VSMC-specific BCAT2 deficiency inhibited diabetic atherosclerotic calcification without sex bias. Furthermore, we revealed that BCKA supplementation reversed VSMC osteoblastic differentiation inhibited by BCAT2 deficiency. Mechanistically, BCAT2-mediated BCKA-derived prop-CoA generation promotes propionylation at lysine 23 of histone H3 (H3K23pr), which contributes to the activation of RUNX2 expression, thereby accelerating VSMC osteoblastic differentiation and VC in diabetic plaques. Taken together, we uncovered the role of BCAT2 in diabetic atherosclerotic calcification and demonstrated its potential as a therapeutic target.

## Results

### Spatial metabolomics profiling identifies enhanced BCAA catabolism in calcified anterior tibial arteries from diabetic foot amputation

**Fig. 1.**
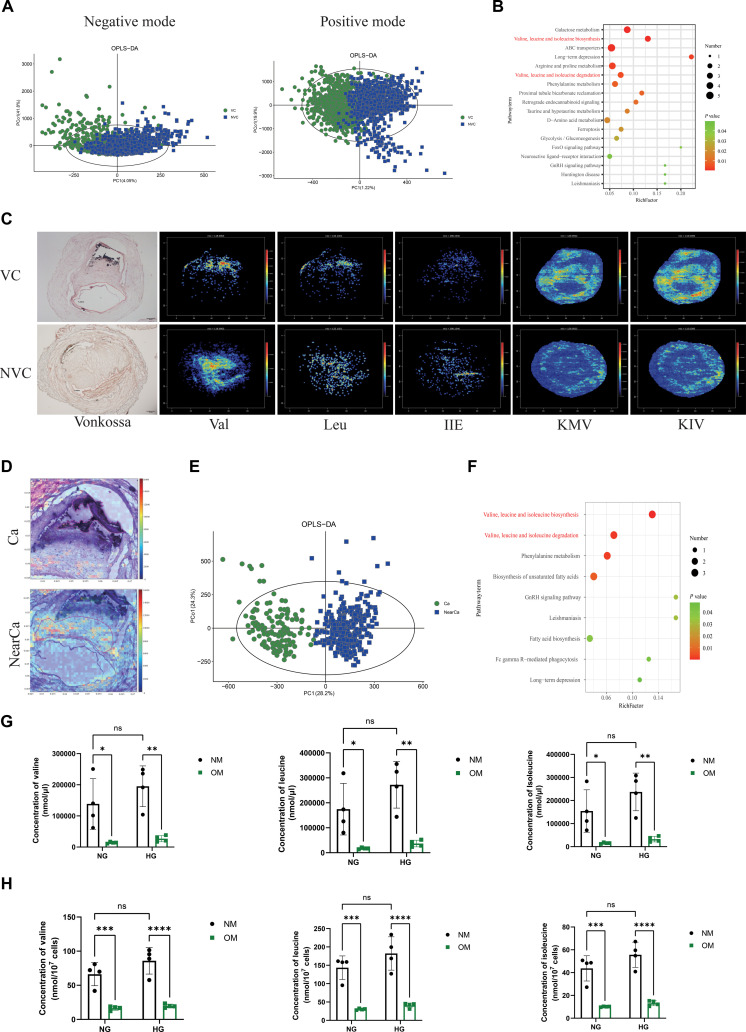
Altered BCAA catabolism in calcification of diabetic plaque. (A) Orthogonal partial least squares discriminant analysis (OPLS-DA) models based on positive and negative ion mode AFADESI-MSI data. Each dot represented one pixel from MSI. VC samples are displayed by green dots. NVC samples are represented by blue dots. Goodness of fit *R*^2^ (*X*) = 0.775, *R*^2^ (*Y*) = 0.234, goodness of prediction Q2 = 0.18. (B) Kyoto Encyclopedia of Genes and Genomes (KEGG) pathway enrichment analysis of identified metabolites with variable importance in projection (VIP) value more than 1 between VC and NVC groups. The top 18 enriched pathways are shown. (C) Left panel: Adjacent von Kossa-stained tissue images. Scale bar, 200 μm. Right panel: Array of MS images of BCAA pathway metabolites from VC (above) and NVC (below) groups by airflow-assisted desorption electrospray ionization mass spectrometry imaging (AFADESI-MSI. (D) VC tissue section zoomed in to show Ca and NearCa group corresponding to the H&E staining and MS image. (E) OPLS-DA score plot showing distribution based on metabolite profiles of Ca and NearCa groups. Each dot represented one pixel from MSI. Ca groups are displayed by green dots. NearCa groups are represented by blue dots. Goodness of fit *R*^2^ (*X*) = 0.678, *R*^2^ (*Y*) = 0.845, goodness of prediction Q2 = 0.815. (F) KEGG pathway enrichment analysis of identified metabolites with VIP value more than 1 between Ca and NearCa groups. The top 9 enriched pathways are shown. (G) Concentration of valine, leucine, and isoleucine in Movas treated with normal media (NM) or osteogenic media (OM) under normal glucose (NG) or high-glucose (HG) treatment for 14 d. *n* = 4. (H) Concentration of valine, leucine, and isoleucine in cell supernatant. Data are presented as mean ± SD. *P < 0.05, **P < 0.01, ***P < 0.001, and ****P < 0.0001 versus NM. ns, not significant.

Our previous study performed spatial metabolomics analyses on frozen sections of anterior tibial arteries collected from individuals subjected to diabetic foot amputation [23]. The sections were divided into no VC (NVC) and VC groups by hematoxylin and eosin (H&E) staining and computed tomography (CT) of lower limbs (Fig. S1A). Compared with the NVC group, the glucose concentration in the VC group decreased, while the levels of pyruvate and lactate increased (Fig. S1B). The reason for this is glucose metabolism reprogramming in calcified anterior tibial artery of diabetic foot amputation. Furthermore, compared with the NVC group, the levels of fatty acids (FAs) and acetone in the VC group increased, suggesting that FAs cannot be effectively utilized and numerous ketone bodies are generated in the calcified anterior tibial artery (Fig. S1C). To further analyze the MSI data, 1,000 pixels were selected from each artery section of both groups. The resulting metabolic profiles were clearly segregated between the NVC and VC groups based on orthogonal partial least squares discriminant analysis (OPLS-DA) analysis (Fig. 1A). Furthermore, metabolic pathway analysis using the Kyoto Encyclopedia of Genes and Genomes (KEGG) library identified BCAA biosynthesis and degradation metabolism as marked enrichment among the altered metabolites (Fig. 1B).

Metabolic enzymes, serving as crucial nodes in the biological metabolic network, connect and regulate complex metabolic reactions and can act as potential drug targets [24]. Therefore, the expression of key metabolic enzymes in the top 6 enriched metabolic pathways was detected by immunohistochemical staining. Galactose mutarotase (GALM) is a key enzyme in galactose metabolism. BCAT1/BCAT2 catalyzes the synthesis and catabolic pathways of BCAA. ABCA1 is a member of the adenosine triphosphate (ATP)-binding cassette (ABC) transporter superfamily. Phospholipase A2 (PLA2) catalyzes glutamate to arachidonic acid. Guanidinoacetate *N*-methyltransferase (GAMT) uses *S*-adenosylmethionine as a methyl donor to convert guanidinoacetate into creatine. As shown in Fig. S2A, compared with the NVC group, the expression of BCAT2, a key metabolic enzyme in the catabolism of BCAA, changed most significantly in the VC group.

Interestingly, among all the identified metabolites involved in the BCAA pathway, valine, leucine, and isoleucine decreased, while KMV and KIV increased, in calcified anterior tibial artery by mass spectrometry (MS) images (Fig. 1C). Further analysis of the spatial changes in metabolism profiling with VC groups, the precise regions of the spatial metabolome were divided into calcification (Ca) and near calcification (NearCa) by H&E staining (Fig. 1D). The resulting metabolic profiles were clearly segregated between the Ca and NearCa groups based on OPLS-DA analysis (Fig. 1E). Strikingly, pathway analysis identified BCAA metabolism as the greatest enrichment among the altered metabolites (Fig. 1F). Next, targeted metabolomics were used to detect the concentration of valine, leucine, and isoleucine in cells and cell supernatants. The results showed that, both in the high-glucose (HG) environment of cells and cell supernatants, the osteogenic medium (OM) group showed a decrease in valine, leucine, and isoleucine compared to the normal medium (NM) group, and the same changes were observed in normal glucose (NG) environment (Fig. 1G and H). These results indicated that BCAA catabolism is enhanced and maintained by increasing BCAA uptake during diabetic atherosclerotic calcification.

### BCAT2 is highly expressed in VSMCs during diabetic atherosclerotic calcification

**Fig. 2.**
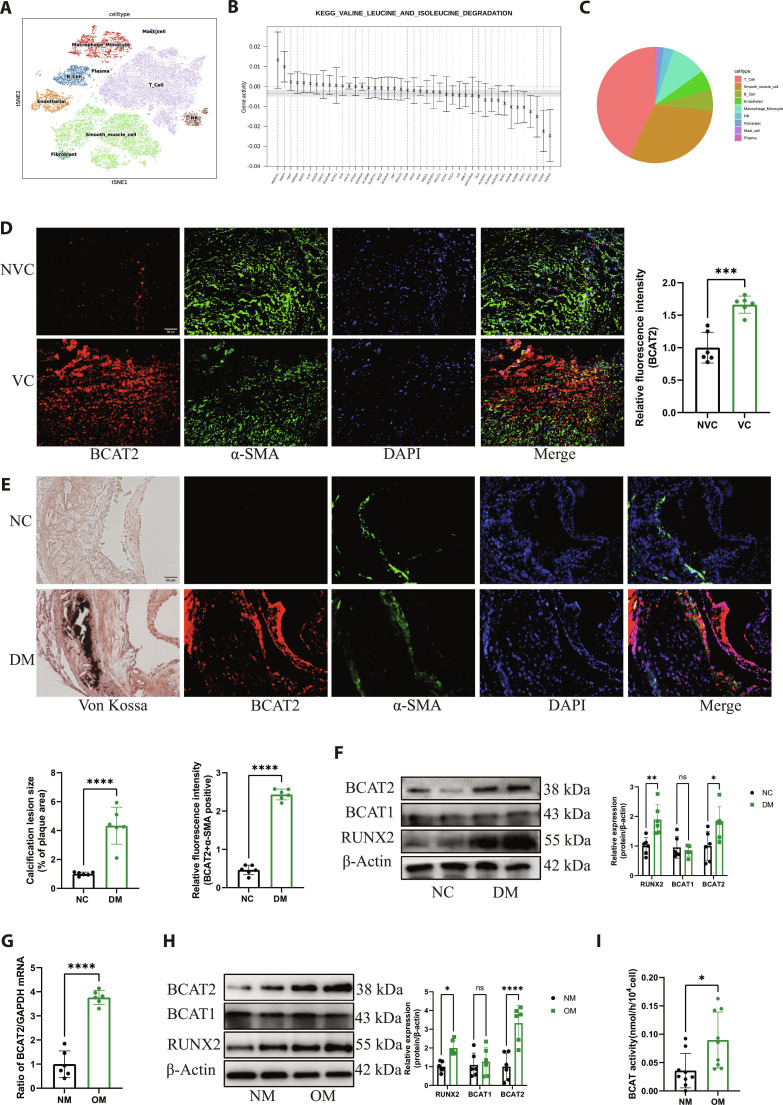
BCAT2 is markedly increased in VSMCs during diabetic atherosclerotic calcification. (A) *t*-Distributed stochastic neighbor embedding (t-SNE) showing the main cell types of anterior tibial arteries collected from individuals subjected to diabetic foot amputation. Population identities were determined based on marker gene expression. (B) Box plot showing the activity level of each gene in BCAA catabolism by Qusage analysis. The horizontal axis represents gene names, and the vertical axis represents gene activity. An activity level greater than zero indicates that the corresponding gene is up-regulated in the VC group. On the contrary, the expression is down-regulated. Blue dashed line represents the overall activity of the gene set, and gray box represents the confidence interval of the gene set. (C) Pie graphs showing the expression distribution of BCAT2 among the different subgroups in the cells of anterior tibial arteries collected from individuals subjected to diabetic foot amputation. (D) Representative images and quantification of immunofluorescence staining of BCAT2 and VSMC marker α-SMA in anterior tibial arteries from diabetic foot amputation. Red, BCAT2; green, α-SMA; blue, DAPI. Scale bar, 50 μm. *n* = 6. ***P < 0.001 versus NVC. (E) Left panel: Adjacent von Kossa-stained tissue images. Right panel: Representative images of immunofluorescence staining of BCAT2 and α-SMA in the aorta of NC or DM ApoE^−/−^ mice. Red, BCAT2; green, α-SMA; blue, DAPI. Scale bar, 50 μm. *n* = 6. ****P < 0.0001 versus NC. (F) Western blot image and analysis showing RUNX2, BCAT1, and BCAT2 protein expression level in the aorta of NC or DM ApoE^−/−^ mice. *n* = 6. *P < 0.05, **P < 0.01 versus NC. (G) Quantitative reverse transcription polymerase chain reaction (qRT-PCR) analysis of BCAT2 gene expression in Movas treated with NM or OM for 14 d. *n* = 6. ****P < 0.0001 versus NM. (H) Western blot image and analysis showing RUNX2, BCAT1, and BCAT2 protein expression level in Movas treated with NM or OM for 14 d. *n* = 6. *P < 0.05, ****P < 0.0001 versus NM. (I) BCAT2 enzyme activity in Movas treated with NM or OM for 14 d. *n* = 9. *P < 0.05 versus NM. Data are presented as mean ± SD.

Our previous study performed single-cell RNA sequencing (scRNA-seq) analyses on anterior tibial arteries collected from individuals subjected to diabetic foot amputation [23]. The samples were divided into NVC and VC groups by CT of lower limbs. Cluster analysis was used for filtered cells, resulting in 27 distinct clusters. By assessing the presence and abundance of canonical cell signature genes within each cluster, we identified 9 cell clusters. The clusters were identified to be T cell (CD3D, CD3E, and CD3G), natural killer cell (NKG7, KLRD1, and KLRK1), B cell (CD79A, CD79B, and CD19), plasma (JCHAIN), macrophage/monocyte cell (HLA-DRA, C1QA, and C1QB), mast cell (MS4A2), smooth muscle cell (ACTA2, MYH11, and MYL9), endothelial cell (EC) (PECAM1, CDH5, and VWF), and fibroblast (COL1A1, COL1A2, and DCN) (Fig. 2A). Gene activity is a quantitative gene enrichment analysis strategy that utilizes quantitative of gene set expression activation (Qusage) analysis to analyze gene sets and gene expression, displaying the activity levels of each gene in the gene set. Qusage analysis identified that BCAT2 is the most active gene in the rate-limiting enzyme of BCAA catabolism pathway (Fig. 2B). We further analyzed the expression pattern of BCAT2 among the different subgroups. The results showed that BCAT2 was mainly expressed in smooth muscle cell and T cell (Fig. 2C).

Given that macrophage cell (Mø), VSMC, and EC are the primary cell for VC, we examined BCAT2 protein expression in 3 cells. The results showed that the expression of BCAT2 had the most significant difference between the NM and OM group in VSMC (Fig. S2B). Furthermore, the costaining of BCAT2 and VSMC marker α-smooth muscle actin (α-SMA), macrophage cell marker CD68, and EC marker CD31 showed that BCAT2 was localized in α-SMA-labeled VSMC in calcified anterior tibial arteries from diabetic foot amputation, consistent with the results of scRNA-seq analysis (Fig. 2D and Fig. S2C). In addition, as shown in Fig. 2E, the colocalization of BCAT2 and α-SMA was increased in the aortic root of diabetic male ApoE^−/−^ mice. Compared with the normal control (NC) group, the BCAT2 protein level was significantly increased in the diabetes mellitus (DM) group, but the BCAT1 protein level had no significant change (Fig. 2F). Consistently, compared to NM-treated Movas, BCAT2 mRNA, protein levels, and enzymatic activity were significantly increased in OM-treated mouse aortic smooth muscle cells (Movas) (Fig. 2G to I).

To explore the underlying molecular mechanisms of diabetic atherosclerotic calcification-induced BCAT2 up-regulation, we identified 2 transcription factors by combining single-cell regulatory network inference analysis (SCENIC) and PROMO tools, including activating transcription factor 3 (ATF3) and MYC-associated zinc finger protein (MAZ) (Fig. S3A). Furthermore, quantitative reverse transcription polymerase chain reaction (qRT-PCR) showed that ATF3 expression was up-regulated in the OM group compared to the NM group, while the change in MAZ expression was not statistically significant (Fig. S3B). At the same time, Western blot indicated an increase in ATF3 protein expression in the OM group (Fig. S3C). Immunofluorescence staining showed nuclear localization of ATF3 and BCAT2, with ATF3 entering the nucleus more significantly in the OM group than in the NM group (Fig. S3D). Chromatin immunoprecipitation (ChIP) assays confirmed that compared with the immunoglobulin G (IgG) group, ATF3 was significantly enriched at the BCAT2 promoter site (Fig. S3E). Interestingly, luciferase reporter gene assays showed that overexpression of ATF3 can inhibit the activity of BCAT2 (Fig. S3F). To determine the transcriptional regulation of BCAT2 binding sites by ATF3, 4 truncated forms of the promoter region (Del1, Del2, Del3, and Del4) were constructed at the position of the promoter region. The fluorescence intensity of Del3 was not significantly different from that of wild type (WT), while the fluorescence intensities of Del1, Del2, and Del4 were significantly higher than that of WT, indicating that the binding site of ATF3 is between BCAT2 promoter −1,000 and −500 base pairs (bp) (Fig. S3G). It is interesting to note that knockdown of ATF3 significantly increased BCAT2 mRNA and protein expression (Fig. S3H and I), suggesting that this transcription factor inhibits, rather than stimulates, BCAT2 expression. Furthermore, the binding efficiency of ATF3 to the BCAT2 promoter was decreased after high-glucose treatment (Fig. S3J).

### VSMC-specific BCAT2 deficiency inhibits VSMC osteogenic differentiation and diabetic atherosclerotic calcification

To explore the role of BCAT2 in the formation of calcification in diabetes plaque, 6-week-old male ApoE^−/−^ mice were used to establish a diabetic atherosclerotic calcification model and then divided into the LV-NC group and LV-BCAT2 group (Fig. S4A). Calcium content and alkaline phosphatase (ALP) activity in the aorta of mice were significantly down-regulated after the knockdown of BCAT2 (Fig. S4B and C). Furthermore, alizarin red S staining and micro-CT of the whole aorta showed that knockdown of BCAT2 reduced aortic calcification in diabetic plaque (Fig. S4D and E). After knocking down BCAT2, the expression of α-SMA and SM22α increased significantly, while that of RUNX2 and BMP2 decreased significantly (Fig. S4F). H&E and von Kossa staining of the aortic root and aortic arch sections also suggested that knockdown of BCAT2 could significantly decrease calcification in diabetic plaque (Fig. S4G). These results indicate that knockdown of BCAT2 can inhibit osteogenic differentiation and VC in diabetic ApoE^−/−^ mice.

**Fig. 3.**
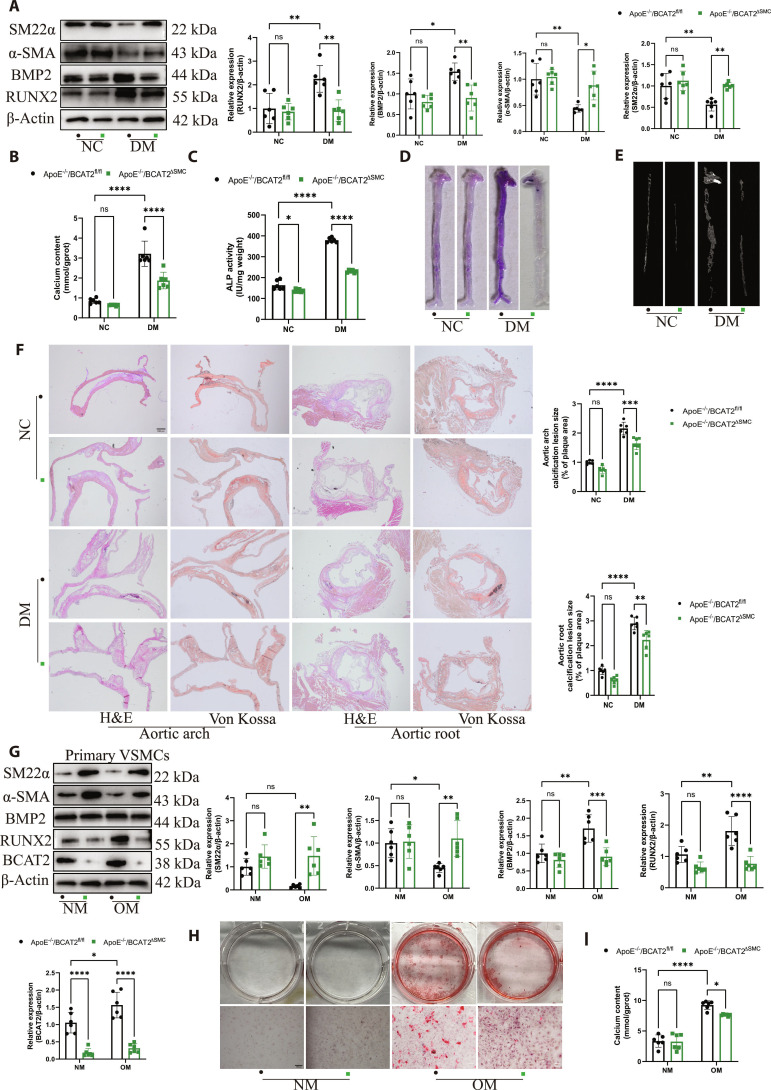
VSMC-specific BCAT2 deficiency inhibits VSMC osteogenic differentiation and diabetic atherosclerotic calcification. (A) Western blot image and analysis showing SM22α, α-SMA, BMP2, and RUNX2 protein expression level in the aorta of ApoE^−/−^/BCAT2^fl/fl^ and ApoE^−/−^/BCAT2^ΔSMC^ mice with NC or DM. *n* = 6. (B and C) Biochemical measurement of aortic calcium content and ALP activity for indicated mice. *n* = 6. (D) Representative images of alizarin red S staining in the whole aorta for indicated mice. (E) Micro-CT was used to detect aortic calcification for indicated mice. (F) Representative images and quantification of H&E and von Kossa staining in aortic arch and aortic root tissue sections. Scale bars, 200 μm. *n* = 6. (G) Western blot image and analysis showing SM22α, α-SMA, BMP2, RUNX2, and BCAT2 protein expression level in VSMCs from ApoE^−/−^/BCAT2^fl/fl^ and ApoE^−/−^/BCAT2^ΔSMC^ mice under NM or OM treatment for 14 d. *n* = 6. (H) Representative images of alizarin red S staining in VSMCs isolated from ApoE^−/−^/BCAT2^fl/fl^ and ApoE^−/−^/BCAT2^ΔSMC^ mice under NM or OM treatment for 14 d. Scale bars, 100 μm. *n* = 6. (I) Quantification of calcium deposits. *n* = 6. Data are presented as mean ± SD. *P < 0.05, **P < 0.01, ***P < 0.001, ****P < 0.0001 versus ApoE^−/−^/BCAT2^fl/fl^.

To further determine whether BCAT2 deficiency in VSMCs may play a critical role in diabetic atherosclerotic calcification, we generated ApoE^−/−^ background mice with a specific deletion of BCAT2 in VSMCs (ApoE^−/−^/BCAT2^fl/fl^Tagln^Cre^ [ApoE^−/−^/BCAT2^ΔSMC^]) and their littermates (ApoE^−/−^/BCAT2^fl/fl^) and then established a diabetic atherosclerotic calcification model (Fig. S5A). The genomic DNA of ApoE^−/−^/BCAT2^ΔSMC^ offspring mice was extracted, the DNA of the offspring mice was amplified by PCR, and the genotype of the offspring mice was identified (Fig. S5B). Immunofluorescence staining demonstrated a significant decrease of BCAT2 protein expression in the ApoE^−/−^/BCAT2^ΔSMC^ mice compared with the ApoE^−/−^/BCAT2^fl/fl^ mice (Fig. S5C). Furthermore, the colocalization of BCAT2 with VSMC-specific marker α-SMA was in ApoE^−/−^/BCAT2^fl/fl^ mice, while there was no BCAT2 expression in VSMCs from ApoE^−/−^/BCAT2^ΔSMC^ mice (Fig. S5D). Western blot showed that the expression of α-SMA and SM22α increased, while that of RUNX2 and BMP2 decreased in the aorta of male ApoE^−/−^/BCAT2^ΔSMC^ mice compared with their littermates (Fig. 3A). Moreover, the aorta of male ApoE^−/−^/BCAT2^ΔSMC^ mice had lower levels of calcium content and ALP activity than that of male ApoE^−/−^/BCAT2^fl/fl^ mice (Fig. 3B and C). As expected, alizarin red S staining and micro-CT analysis also revealed that VSMC-specific BCAT2 deficiency reduced aortic calcification in diabetic plaque (Fig. 3D and E), and this result was further confirmed by H&E and von Kossa of the aortic root and aortic arch sections (Fig. 3F). Taken together, these findings suggest that VSMC-specific BCAT2 deficiency inhibits VSMC osteogenic differentiation and diabetic atherosclerotic calcification.

To demonstrate the effect of sex-based differences in diabetic atherosclerotic calcification, we fed female ApoE^−/−^/BCAT2^fl/fl^ and ApoE^−/−^/BCAT2^ΔSMC^ mice, and established a diabetic atherosclerotic calcification model. We found that the expression of α-SMA and SM22α increased, while that of RUNX2 and BMP2 decreased in the aorta from female VSMC-specific BCAT2 deficiency mice (Fig. S6A). The aorta of female ApoE^−/−^/BCAT2^ΔSMC^ mice had lower levels of calcium content and ALP activity than that of female ApoE^−/−^/BCAT2^fl/fl^ mice (Fig. S6B and C), along with decreased aortic calcification revealed by H&E and von Kossa staining, which matched the results of male ApoE^−/−^/BCAT2^ΔSMC^ mice (Fig. S6D). Taken together, our findings suggest that VSMC-specific BCAT2 deficiency alleviates diabetic atherosclerotic calcification formation without sex bias.

To demonstrate the in vivo findings, VSMCs were isolated from ApoE^−/−^/BCAT2^fl/fl^ and ApoE^−/−^/BCAT2^ΔSMC^ mice and treated with NM or OM for 14 d under a high-glucose and high-fat environment. VSMCs from ApoE^−/−^/BCAT2^ΔSMC^ mice had markedly decreased RUNX2 and BMP2 expression but increased α-SMA and SM22α expression (Fig. 3G). Alizarin red S staining and calcium content detection also suggested that VSMC-specific BCAT2 deficiency could significantly decrease VSMC calcification (Fig. 3H and I). We further confirmed that knockdown of BCAT2 had significantly reduced RUNX2 and BMP2 expression but increased α-SMA and SM22α expression (Fig. S7A). Alizarin red S staining and calcium content detection also suggested that knockdown of BCAT2 decreased Movas calcification (Fig. S7B and C). Moreover, we found that overexpression of BCAT2 increased RUNX2 and BMP2 expression but decreased α-SMA and SM22α expression (Fig. S8A). Alizarin red S staining and calcium content detection also suggested that overexpression of BCAT2 increased Movas calcification (Fig. S8B and C).

### BCKA supplementation reverses VSMC osteogenic differentiation and diabetic atherosclerotic calcification inhibited by BCAT2 deficiency

**Fig. 4.**
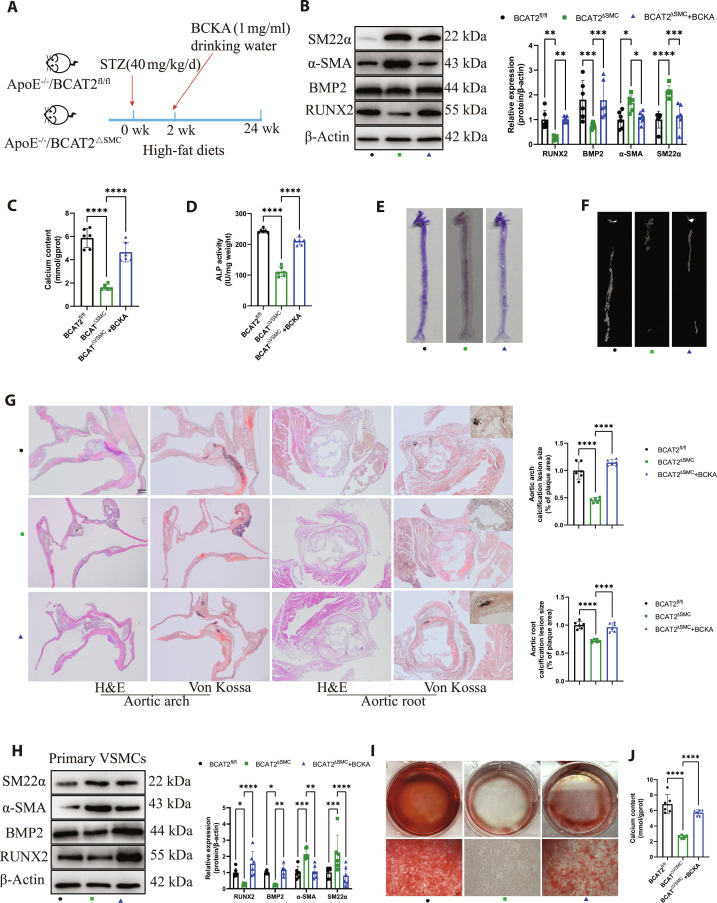
BCKA supplement promotes VSMC osteogenic differentiation and diabetic atherosclerotic calcification. (A) Schematic protocol: ApoE^−/−^/BCAT2^fl/fl^ and ApoE^−/−^/BCAT2^ΔSMC^ mice were intraperitoneally injected with streptozotocin (STZ) (40 mg/kg) for 5 d and then supplemented with 1 mg/ml BCKA in drinking water together with the high-fat diet for 24 weeks. (B) Western blot image and analysis showing SM22α, α-SMA, BMP2, and RUNX2 protein expression level in the aorta of ApoE^−/−^/BCAT2^fl/fl^, ApoE^−/−^/BCAT2^ΔSMC^, and ApoE^−/−^/BCAT2^ΔSMC^ supplemented with 1 mg/ml BCKA in drinking water. *n* = 6. (C and D) Biochemical measurement of aortic calcium content and ALP activity for indicated mice. *n* = 6. (E) Representative images of alizarin red S staining in the whole aorta for indicated mice. (F) Micro-CT was used to detect aortic calcification for indicated mice. (G) Representative images and quantification of H&E and von Kossa staining in aortic arch and aortic root tissue sections. Scale bars, 200 μm. *n* = 6. (H) Western blot image and analysis showing SM22α, α-SMA, BMP2, and RUNX2 protein expression level in VSMCs from ApoE^−/−^/BCAT2^fl/fl^ and ApoE^−/−^/BCAT2^ΔSMC^ supplemented with or without BCKA under OM treatment for 14 d. *n* = 6. (I) Representative images of alizarin red S staining in VSMCs from ApoE^−/−^/BCAT2^fl/fl^ and ApoE^−/−^/BCAT2^ΔSMC^ supplemented with or without BCKA under OM treatment for 14 d. Scale bars, 200 μm. *n* = 6. (J) Quantification of calcium deposits. *n* = 6. Data are presented as mean ± SD. *P < 0.05, **P < 0.01, ***P < 0.001, ****P < 0.0001 versus ApoE^−/−^/BCAT2^fl/fl^ or ApoE^−/−^/BCAT2^ΔSMC^.

To identify the role of BCAT2-mediated BCAA catabolism in diabetic atherosclerotic calcification, we added BCKA of BCAT2-catabolized BCAA degradation to the drinking water of diabetic ApoE^−/−^/BCAT2^ΔSMC^ mice and fed with high-fat diet (HFD) for 24 weeks. After BCKA supplementation, the expression of α-SMA and SM22α decreased in the aorta of diabetic ApoE^−/−^/BCAT2^ΔSMC^ mice, while the expression of RUNX2 and BMP2 significantly increased (Fig. 4A). The ApoE^−/−^/BCAT2^ΔSMC^ + BCKA group had higher levels of calcium content and ALP activity in the aorta than the ApoE^−/−^/BCAT2^ΔSMC^ group (Fig. 4B and C). As expected, alizarin red S staining and micro-CT analysis of aorta also revealed that BCKA supplementation increased aortic calcification (Fig. 4D and E), and this result was further confirmed by H&E and von Kossa of the aortic root and aortic arch sections (Fig. 4F). Furthermore, we incubated primary VSMCs isolated from ApoE^−/−^/BCAT2^ΔSMC^ mice under OM treatment for 14 d, supplemented with 0.6 mM BCKA. Western blot showed that BCKA supplementation had significantly increased RUNX2 and BMP2 expression but decreased α-SMA and SM22α expression (Fig. 4G). Alizarin red S staining and calcium content detection also suggested that BCKA supplementation increased VSMC calcification (Fig. 4H and I).

Moreover, we investigated the effect of BCAA on the formation of diabetic atherosclerotic calcification. H&E and von Kossa staining showed that compared with the NC group, calcification in diabetes plaque of aortic root and aortic arch in high BCAA group increased (Fig. S9A). High BCAA diet can significantly increase calcium content and ALP activity in the aorta of diabetic ApoE^−/−^ mice (Fig. S9B and C). Next, we incubated Movas under OM treatment for 14 d, supplemented with 800 or 1,600 μM BCAA. Alizarin red S staining suggested that high concentrations of BCAA can significantly increase Movas calcification, and the calcium content detection is consistent with the results of alizarin red S staining (Fig. S9D and E). The above results indicate that high concentration of BCAA can promote diabetic atherosclerotic calcification.

### KMV or KIV supplementation, but not KIC, promotes VSMC osteogenic differentiation and diabetic atherosclerotic calcification

**Fig. 5.**
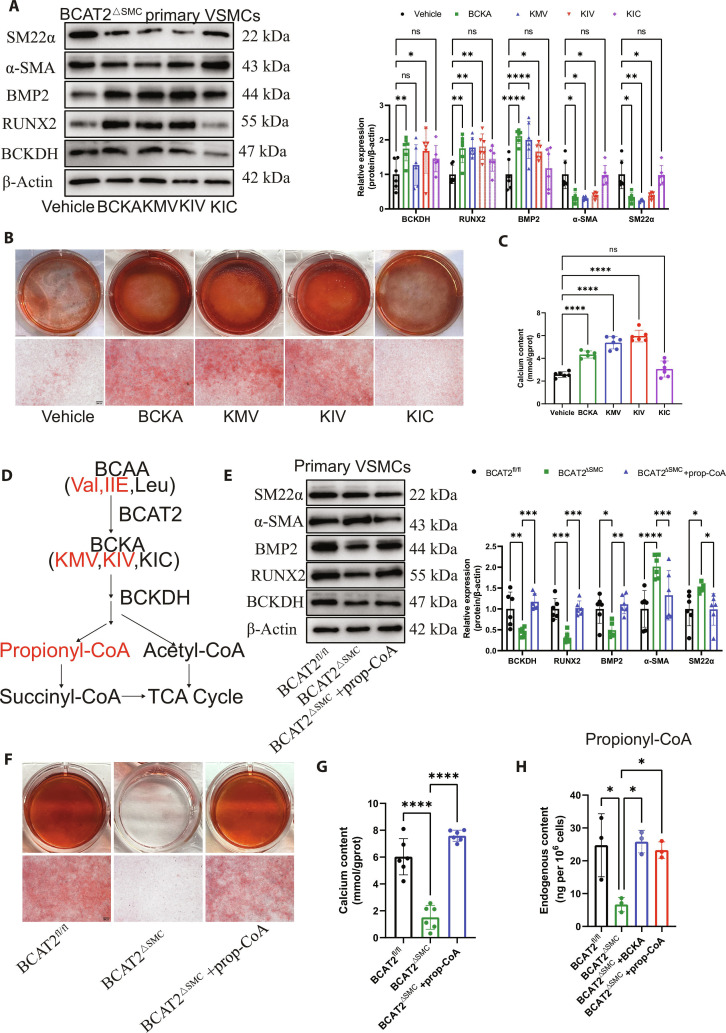
KMV or KIV supplement, but not KIC, promotes VSMC osteogenic differentiation and diabetic atherosclerotic calcification. (A) Western blot image and analysis showing BCKDH, SM22α, α-SMA, BMP2, and RUNX2 protein expression level in VSMCs from ApoE^−/−^/BCAT2^ΔSMC^ mice supplemented with vehicle, BCKA, KMV, KIV, or KIC for 72 h under OM treatment for 14 d. *n* = 6. *P < 0.05, **P < 0.01, ***P < 0.001, ****P < 0.0001 versus vehicle. (B) Representative images of alizarin red S staining in VSMCs from ApoE^−/−^/BCAT2^ΔSMC^ mice supplemented with vehicle, 0.6 mM BCKA, 0.6 mM KMV, 0.6 mM KIV, or 0.6 mM KIC for 72 h under OM treatment for 14 d. Scale bars, 200 μm. *n* = 6. (C) Quantification of calcium deposits. *n* = 6. ****P < 0.0001 versus vehicle. (D) Schematic map for BCAA catabolism pathway. BCAA contains valine, leucine, and isoleucine. BCKA contains KMV, KIC, and KIV. (E) Western blot image and analysis showing BCKDH, SM22α, α-SMA, BMP2, and RUNX2 protein expression level in VSMCs from ApoE^−/−^/BCAT2^ΔSMC^ mice supplemented with or without prop-CoA under OM treatment for 14 d. *n* = 6. *P < 0.05, ***P < 0.001, ****P < 0.0001 versus ApoE^−/−^/BCAT2^fl/fl^ or ApoE^−/−^/BCAT2^ΔSMC^. (F) Representative images of alizarin red S staining in VSMCs from ApoE^−/−^/BCAT2^ΔSMC^ mice supplemented with or without prop-CoA under OM treatment for 14 d. Scale bars, 200 μm. *n* = 6. (G) Quantification of calcium deposits. *n* = 6. ****P < 0.0001 versus ApoE^−/−^/BCAT2^fl/fl^ or ApoE^−/−^/BCAT2^ΔSMC^. (H) Quantification of prop-CoA in VSMCs from ApoE^−/−^/BCAT2^fl/fl^ and ApoE^−/−^/BCAT2^ΔSMC^ mice with vehicle, BCKA, or prop-CoA under OM treatment for 14 d. *n* = 3. *P < 0.05 versus ApoE^−/−^/BCAT2^fl/fl^ or ApoE^−/−^/BCAT2^ΔSMC^. Data are presented as mean ± SD.

BCAT2 converts BCAA to BCKA containing KIV, KIC, and KMV, and BCKDH degrades KMV and KIV to prop-CoA while degrading KMV and KIC to acetyl-CoA [17]. Interestingly, Western blot showed that KMV or KIV supplementation inhibits the expression of α-SMA and SM22α while promoting the expression of BCKDH, RUNX2, and BMP2, where the effect of KIC is not statistically significant (Fig. 5A). Alizarin red S staining and calcium content detection also suggested that KMV or KIV supplementation increased VSMC calcification, while the supplementation of KIC had minor effects (Fig. 5B and C). Moreover, high concentrations of valine and isoleucine promote Movas calcification, while leucine has no significant effects (Fig. S9F and G). Especially, KMV and KIV share a similar metabolic pathway metabolized into prop-CoA (Fig. 5D). Prop-CoA supplementation inhibited the expression of α-SMA and SM22α while promoting the expression of BCKDH, RUNX2, and BMP2 (Fig. 5E). Alizarin red S staining and calcium content detection also suggested that prop-CoA supplementation enhanced VSMC calcification (Fig. 5F and G). Consistently, prop-CoA concentration was significantly decreased in ApoE^−/−^/BCAT2^ΔSMC^ mice compared to ApoE^−/−^/BCAT2^fl/fl^ mice, while exogenous supplementation of BCKA or prop-CoA increased the concentration of prop-CoA (Fig. 5H). However, the change of acetyl-CoA or succinyl-CoA concentration had no statistical significance after VSMC-specific BCAT2 deficiency (Fig. S10A and B).

### VSMC-specific BCAT2 deficiency inhibits histone propionylation

**Fig. 6.**
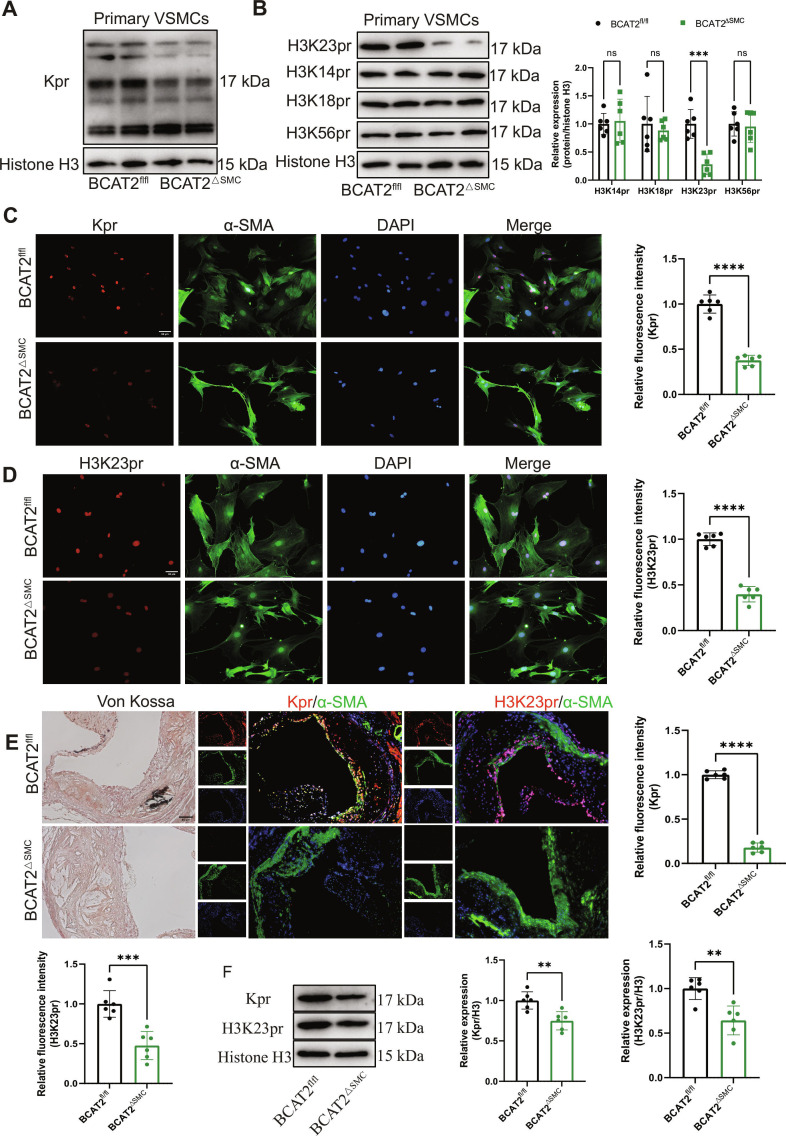
VSMC-specific BCAT2 deficiency inhibits histone propionylation. (A) Western blot images and analysis show protein propionylation levels in VSMCs from ApoE^−/−^/BCAT2^fl/fl^ and ApoE^−/−^/BCAT2^ΔSMC^ mice under OM treatment for 14 d. *n* = 6. (B) Western blot image and analysis showing H3K23pr, H3K14pr, H3K18pr, and H3K56pr protein expression levels in VSMCs from ApoE^−/−^/BCAT2^fl/fl^ and ApoE^−/−^/BCAT2^ΔSMC^ mice under OM treatment for 14 d. *n* = 6. (C) Representative images and quantification of immunofluorescence staining of Kpr and α-SMA in VSMCs from ApoE^−/−^/BCAT2^fl/fl^ and ApoE^−/−^/BCAT2^ΔSMC^ mice under OM treatment for 14 d. Red, Kpr; green, α-SMA; blue, DAPI. Scale bar, 50 μm. *n* = 6. (D) Representative images and quantification of immunofluorescence staining of H3K23pr and α-SMA in VSMCs from ApoE^−/−^/BCAT2^fl/fl^ and ApoE^−/−^/BCAT2^ΔSMC^ mice under OM treatment for 14 d. Red, H3K23pr; green, α-SMA; blue, DAPI. Scale bar, 50 μm. *n* = 6. (E) Left panel: Adjacent von Kossa-stained tissue images. Medium panel: Representative images of immunofluorescence staining of Kpr and α-SMA in the aorta of ApoE^−/−^/BCAT2^fl/fl^ and ApoE^−/−^/BCAT2^ΔSMC^ mice. Right panel: Representative images of immunofluorescence staining of H3K23pr and α-SMA in the aorta of ApoE^−/−^/BCAT2^fl/fl^ and ApoE^−/−^/BCAT2^ΔSMC^ mice. Red, Kpr/H3K23pr; green, α-SMA; blue, DAPI. Scale bar, 50 μm. *n* = 6. (F) Western blot images and analysis show protein propionylation and H3K23pr protein expression levels in the aorta of ApoE^−/−^/BCAT2^fl/fl^ and ApoE^−/−^/BCAT2^ΔSMC^ mice. Data are presented as mean ± SD. *P < 0.05, **P < 0.01, ***P < 0.001, ****P < 0.0001 versus ApoE^−/−^/BCAT2^fl/fl^.

Propionylation depends on the BCAA-derived prop-CoA and regulates gene expression and disease progression [25]. To investigate the mechanism of prop-CoA in modulating diabetic atherosclerotic calcification, we assessed the levels of protein propionylation in primary VSMCs isolated from ApoE^−/−^/BCAT2^fl/fl^ and ApoE^−/−^/BCAT2^ΔSMC^ mice.Marked decreased protein propionylation in VSMCs from ApoE^−/−^/BCAT2^ΔSMC^ mice compared with ApoE^−/−^/BCAT2^fl/fl^ mice is shown in Fig. 6A. Notably, the changes were more significant around histone locations, which prompted us to focus on alterations in histone propionylation (Kpr). Furthermore, the level of histone 3 lysine 23 site propionylation (H3K23pr) was decreased in VSMCs from ApoE^−/−^/BCAT2^ΔSMC^ mice compared with ApoE^−/−^/BCAT2^fl/fl^ mice, whereas no significant difference in the protein levels of histone 3 lysine 14 site propionylation (H3K14pr), histone 3 lysine 18 site propionylation (H3K18pr), and histone 3 lysine 56 site propionylation (H3K56pr) was observed (Fig. 6B). This finding was further supported by immunofluorescence staining (Fig. 6C and D). To validate in vitro results, the aortic protein propionylation of ApoE^−/−^ BCAT2^fl/fl^ and ApoE^−/−^ BCAT2^ΔSMC^ was detected. Immunofluorescence staining showed that VSMC-specific BCAT2 deficiency reduced calcification in diabetic plaque, as well as decreased Kpr and H3K23pr levels (Fig. 6E). Western blot results were consistent. Compared with ApoE^−/−^ BCAT2^fl/fl^ mice, the level of Kpr and H3K23pr decreased in the aorta of ApoE^−/−^ BCAT2^ΔSMC^ mice (Fig. 6F). Furthermore, both immunofluorescence staining and Western blot showed that knockdown of BCAT2 in Movas decreased Kpr and H3K23pr levels (Fig. S11A to C). Moreover, exogenous supplementation of prop-CoA reversed the H3K23pr protein levels inhibited by VSMC-specific BCAT2 deficiency (Fig. S11D).

We also evaluated Kpr and H3K23pr expression in anterior tibial arteries from diabetic foot amputation. The clinical characteristics of individuals involved in our study were listed in Table S1. The levels of Kpr and H3K23pr were markedly elevated in the VC group than in the NVC group (Fig. S12A and B). Consistently, the levels of Kpr and H3K23pr in Movas treated with OM for 14 d were significantly raised (Fig. S12C to E). Taken together, these results provide compelling evidence that H3K23pr is involved in diabetic atherosclerotic calcification.

### BCAT2–BCKA axis modulates RUNX2-mediated VSMC osteoblastic differentiation and diabetic atherosclerotic calcification

**Fig. 7.**
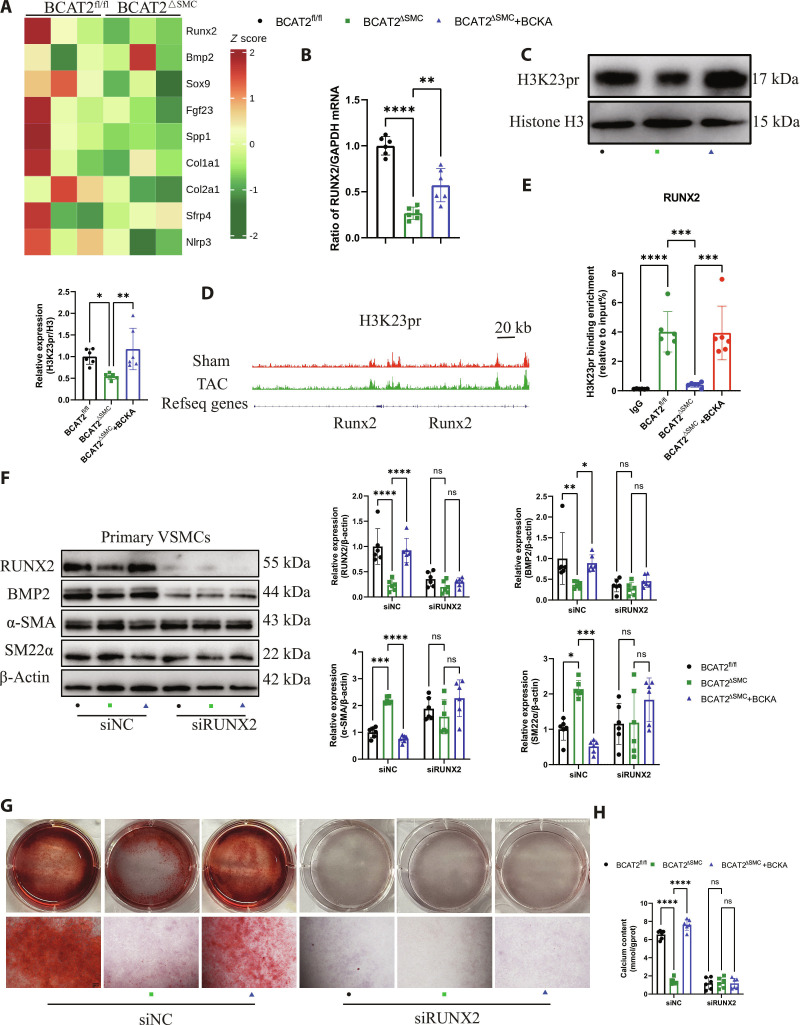
BCAT2–BCKA axis regulates RUNX2 expression via histone propionylation. (A) Heatmap showing the differentially expressed genes of RNA-seq from the aorta of ApoE^−/−^/BCAT2^fl/fl^ and ApoE^−/−^/BCAT2^ΔSMC^ mice in terms of osteoblast differentiation. (B) qRT-PCR analysis of RUNX2 gene expression in VSMCs from ApoE^−/−^/BCAT2^fl/fl^ and ApoE^−/−^/BCAT2^ΔSMC^ supplemented with or without BCKA under OM treatment for 14 d. *n* = 6. (C) Western blot images and analysis show H3K23pr protein expression levels in VSMCs from ApoE^−/−^/BCAT2^fl/fl^ and ApoE^−/−^/BCAT2^ΔSMC^ supplemented with or without BCKA under OM treatment for 14 d. *n* = 6. (D) Genome browser tracks of the ChIP-seq signals from GSE229131 at representative H3K23pr peaks at the RUNX2 locus. Scale bar, 20 kb. (E) ChIP-qPCR assay of H3K23pr in the RUNX2 locus in VSMCs from ApoE^−/−^/BCAT2^fl/fl^ and ApoE^−/−^/BCAT2^ΔSMC^ supplemented with or without BCKA under OM treatment for 14 d. *n* = 6. (F) Western blot image and analysis showing SM22α, α-SMA, BMP2, and RUNX2 protein expression levels in VSMCs transfected with negative control siRNA (siNC) or RUNX2 siRNA (siRUNX2) for 48 h followed by supplementation with or without BCKA for 72 h under OM treatment for 14 d. *n* = 6. (G) Representative images of alizarin red S staining. Scale bars, 200 μm. *n* = 6. (H) Quantification of calcium deposits. *n* = 6. Data are presented as mean ± SD. *P < 0.05, **P < 0.01, ***P < 0.001, ****P < 0.0001 versus ApoE^−/−^/BCAT2^fl/fl^ or ApoE^−/−^/BCAT2^ΔSMC^.

Since the levels of H3K23pr in VSMCs are related to diabetic atherosclerotic calcification, we investigate the mechanisms of BCAT2-mediated BCAA catabolism in modulating diabetic atherosclerotic calcification via histone propionylation. We performed RNA-seq to characterize the transcriptional changes in the aorta of ApoE^−/−^/BCAT2^fl/fl^ and ApoE^−/−^/BCAT2^ΔSMC^ mice. From the RNA-seq analyses, we observed that osteogenic markers in the aorta of ApoE^−/−^/BCAT2^ΔSMC^ mice were down-regulated, including Runx2, Bmp2, Sox9, Fgf23, Spp1, Col1a1, Col2a1, Sfrp4, and Nlrp3, compared with those in the ApoE^−/−^/BCAT2^fl/fl^ mice (Fig. 7A). It has been demonstrated that osteoblastic differentiation of VSMCs is required for atherosclerotic calcification, where RUNX2 is a key osteogenic factor regulating the osteogenic differentiation of VSMCs [20]. Moreover, VSMC-specific BCAT2 deficiency inhibits RUNX2 expression, while further supplementation of BCKA leads to an increase in RUNX2 expression (Fig. 7B). BCKA supplementation restored the down-regulated H3K23pr level in VSMCs isolated from ApoE^−/−^/BCAT2^ΔSMC^ mice (Fig. 7C).

To examine whether RUNX2 mediated VSMC osteoblastic differentiation and diabetic atherosclerotic calcification modulated by the BCAT2–BCKA axis, primary VSMCs were isolated from ApoE^−/−^/BCAT2^fl/fl^ and ApoE^−/−^/BCAT2^ΔSMC^ mice and then transfected with negative control small interfering RNA (siRNA) (siNC) or RUNX2 siRNA (siRUNX2) for 48 h followed by supplementation with or without BCKA for 72 h under OM treatment for 14 d. Western blot revealed that VSMC-specific BCAT2 deficiency decreased the expression of RUNX2 and BMP2, accompanied by the promotion of SM22α and α-SMA, while BCKA supplementation reversed it. However, after knockdown of RUNX2 effectively suppressed VSMC osteogenic differentiation reversed by BCKA supplementation (Fig. 7F). Alizarin red S staining and calcium content detection also suggested that knockdown of RUNX2 markedly inhibited VSMC calcification induced by BCKA supplementation (Fig. 7G and H). Taken together, these results indicate that the BCAT2–BCKA axis modulated VSMC osteogenic differentiation and calcium nodule formation relied on RUNX2 expression mediated by histone propionylation.

Previous studies reported that acetyltransferase P300 possesses propionyltransferase activities [26]. Thus, we further confirmed the role of P300 in histone propionylation. C646 (P300 inhibitor) reduced H3K23pr levelsand RUNX2 and BMP2 expressions induced by BCKA supplementation, while it increased α-SMA and SM22α expressions inhibited by BCKA supplementation (Fig. S13A and B). Alizarin red S staining and calcium content detection also suggested that inhibition of P300 markedly inhibited VSMC calcification induced by BCKA supplementation (Fig. S13C and D).

### BCAT2 inhibitor impedes diabetic atherosclerotic calcification

**Fig. 8.**
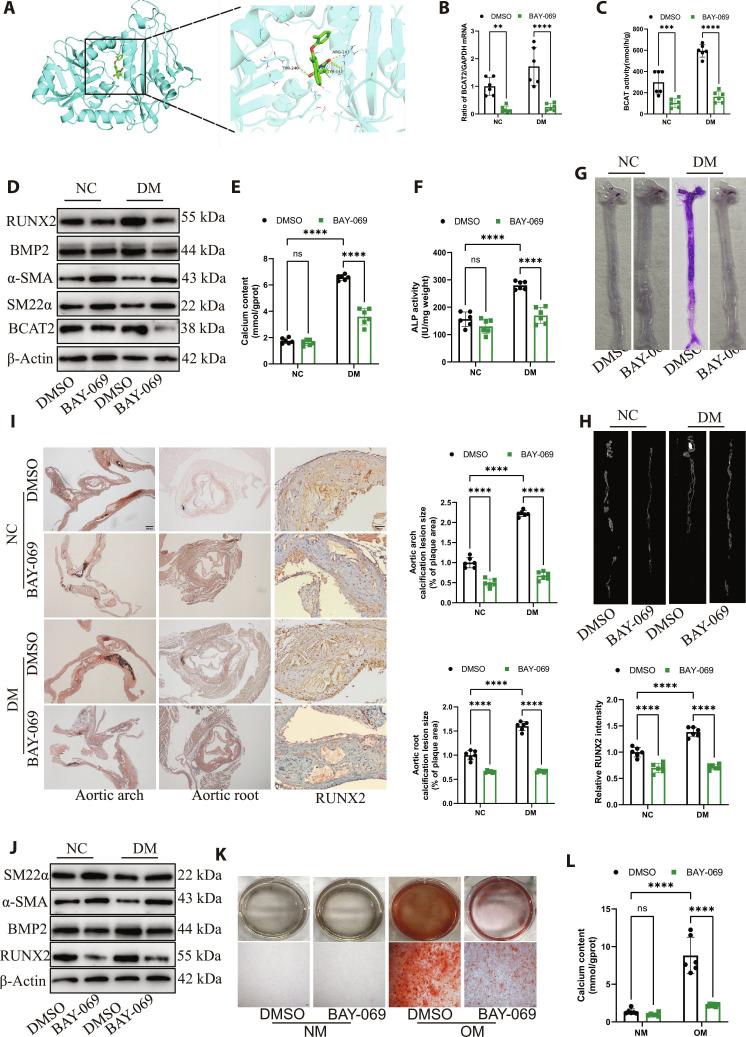
BCAT2 inhibitor impedes diabetic atherosclerotic calcification. (A) Docking model of BAY-069 (BCAT2 inhibitor) and BCAT2. (B) qRT-PCR analysis of BCAT2 gene expression in the aorta of NC or DM ApoE^−/−^ mice supplemented with DMSO or BAY-069 (0.3 mg/kg). *n* = 6. (C) Western blot image and analysis showing α-SMA, SM22α, BMP2, RUNX2, and BCAT2 protein expression level in the aorta of NC or DM ApoE^−/−^ mice supplemented with DMSO or BAY-069 (0.3 mg/kg). *n* = 6. (D) BCAT2 enzyme activity in the aorta of indicated mice. *n* = 6. (E and F) Biochemical measurement of aortic calcium content and ALP activity for indicated mice. *n* = 6. (G) Representative images of alizarin red S staining in the whole aorta for indicated mice. (H) Micro-CT was used to detect aortic calcification for indicated mice. (I) Representative images and quantification of von Kossa staining in aortic arch and aortic root tissue sections, and immunohistochemistry staining of RUNX2 in aortic root tissue sections. Scale bars, 200 μm for von Kossa staining and 50 μm for immunohistochemistry staining. *n* = 6. (J) Western blot image and analysis showing α-SMA, SM22α, BMP2, RUNX2, and BCAT2 protein expression level in Movas supplemented with DMSO or BAY-069 under NM or OM treatment for 14 d. *n* = 6. (K) Representative images of alizarin red S staining. Scale bars, 200 μm. *n* = 6. (L) Quantification of calcium deposits. *n* = 6. Data are presented as mean ± SD. **P < 0.01, ***P < 0.001, ****P < 0.0001 versus DMSO.

Since BCAT2 is closely related to diabetic atherosclerotic calcification development, BAY-069 as a specific inhibitor of BCAT2 was used and assayed the therapeutic effects in treating diabetic arterial calcification. Molecular docking analysis revealed that the potential BAY-069-binding site is located at a surface pocket on BCAT2, including Arg^143^, Thr^240^, and Tyr^141^ (Fig. 8A). qRT-PCR showed that BAY-069 significantly inhibited BCAT2 gene expression (Fig. 8B). Next, enzyme activity analysis showed that BAY-069 significantly inhibited BCAT2 activity (Fig. 8C). Western blot indicated that BAY-069 reduced BCAT2, RUNX2, and BMP2 protein expressions but increased α-SMA and SM22α protein expressions (Fig. 8D and Fig. S14A). Moreover, calcium content and ALP activity in the aorta of diabetic ApoE^−/−^ mice were significantly down-regulated after BAY-069 intervention (Fig. 8E and F). As expected, alizarin red S staining and micro-CT analysis also revealed that aortic calcification in diabetic plaque decreased after BAY-069 intervention (Fig. 8G and H), and this result was further confirmed by H&E and von Kossa of the aortic root and aortic arch sections (Fig. 8I). Consistently, BAY-069 markedly inhibited Movas osteoblastic differentiation and calcium nodule formation (Fig. 8J to L and Fig. S14B). Moreover, the plaque of the whole aorta was significantly decreased in DM ApoE^−/−^ mice treated with BAY-069 compared with the DMSO group (Fig. S15A and B). Furthermore, H&E and oil red O staining showed that plaque lesion area and lipid deposition of the aortic root were significantly decreased in DM ApoE^−/−^ mice treated with BAY-069 compared with the DMSO group (Fig. S15C). These findings suggested that targeting BCAT2 might represent a promising strategy for the treatment of diabetic atherosclerotic calcification.

## Discussion

Multiple metabolic pathways are activated and metabolic reprogramming occurs in the calcified anterior tibial artery with diabetic foot amputation [23]. The emergence of spatial omics technology has advanced the progress of precision treatment for cardiovascular diseases [27]. Here, we have revealed a previously unreported metabolic feature by spatial metabolomics analysis, with enhanced BCAA catabolism in the calcified anterior tibial artery of diabetic foot amputation. Furthermore, BCAT2 is an outstanding metabolic enzyme in the BCAA catabolism pathway and was observed to be highly expressed in VSMCs in calcified anterior tibial arteries from diabetic foot amputation. VSMC-specific BCAT2 deficiency inhibited VSMC osteogenic differentiation and diabetic atherosclerotic calcification, while reversed by BCKA supplementation, especially KMV and KIV supplementation. Mechanistically, VSMC-specific BCAT2 deficiency suppressed the generation of BCKA-derived prop-CoA, mitigating histone propionylation at the promoter of RUNX2, and thereby VSMC osteogenic differentiation and diabetic atherosclerotic calcification. Therefore, our study uncovered a new molecular and metabolic paradigm in the pathogenesis of diabetic atherosclerotic calcification and provided a potential therapeutic target for the disease.

The elevation of plasma BCAA levels is independently associated with the severity of coronary artery lesions [28], while several studies indicated that low plasma BCAA levels were closely related to severe systemic cardiac disease and had an increased risk of death for old men [29–32]. BCAA is not only a substrate for protein synthesis but also a crucial signaling molecule for metabolic regulation. Abnormal catabolism of BCAA participates in the pathogenesis of various diseases such as diabetes, cardiovascular diseases, and cancer by affecting the mammalian target of mammalian target of rapamycin (mTOR) pathway, mitochondrial function, and epigenetic modifications [13]. Based on spatial metabolomics, this study found that the catabolism of BCAA is enhanced in the calcified anterior tibial arteries of diabetic foot amputation. The levels of BCAA decreased, while their catabolite BCKA accumulated in calcified anterior tibial arteries with diabetic foot amputation. Especially, isoleucine and its catabolite KMV had the most significant changes. In fact, catabolic intermediates of BCAA, such as 3-hydroxyisobutyric acid (3-HIB), play a key role in cardiovascular diseases [8,33]. However, the changes of these catabolic intermediates in calcified anterior tibial arteries with diabetic foot amputation are not obvious.

BCAT, including BCAT1 and BCAT2, is a rate-limiting enzyme of BCAA catabolism, mediating the reversible conversion between BCAA and BCKA [8]. In this study, BCAT2 was significantly up-regulated in calcified anterior tibial artery with diabetic foot amputation, while the changes of BCAT1 were not statistically significant. Moreover, scRNA-seq analysis indicated that the expression of BCAT2 was the most significantly up-regulated in the key metabolic enzymes of BCAA catabolism and was mainly distributed in smooth muscle cells. This is consistent with previous studies. BCAT1 is mainly involved in regulating the occurrence and development of cancer, while BCAT2 mainly plays a role in metabolic diseases [13]. Previous studies have shown that high glucose inhibits KLF15 expression, which in turn suppresses the expression of BCAA degrading enzymes such as BCAT2 and BCKDHA, thereby leading to BCAA accumulation [34]. The difference between the 2 studies can be attributed to the different groupings. The conclusion of this study is that the expression of BCAT2 is up-regulated in calcified anterior tibial artery from diabetic foot amputation. ATF3 is a member of the adenosine 3′,5′-monophosphate (cAMP) response element binding family of alkaline leucine zipper TF11, which can bind to gene promoter region and regulate the transcriptional activity [35,36]. Consistent with previous studies [36], ATF3 can act as a negative transcription factor that binds between the −1,000- to −500-bp region of the BCAT2 promoter, thereby inhibiting BCAT2 transcription. In this study, although the compensatory expression of ATF3 is up-regulated in diabetic calcification microenvironment, BCAT2 may be continuously activated by high-glucose signals to override the transcriptional inhibition of ATF3, ultimately leading to the up-regulated expression in diabetic calcification microenvironment. Previous studies have shown that overexpression of KLF15 in cardiomyocytes can induce the expression of BCAA metabolic enzymes [8]. Whether the expression of BCAT2 can be competitively regulated by promoting the expression of KLF15 in diabetic calcified blood vessels to compete with ATF3 requires further investigation in future studies.

Since estrogen has an anti-atherosclerotic effect, gender can affect the formation of atherosclerotic plaques. In males, the plaques are larger and have a higher risk of plaque rupture [37]. In this study, VSMC-specific BCAT2 deficiency inhibits calcification in diabetic atherosclerotic plaques without gender bias. This result suggests that the regulatory effect of BCAT2 on calcification in diabetic plaques is not affected by gender, which eliminates the potential interference of estrogen on calcification formation. Due to the up-regulation of BCAT2, BCAA was significantly reduced and BCKA was elevated in calcified anterior tibial arteries from diabetic foot amputation. More importantly, BCKA supplementation reversed VSMC-specific BCAT2 deficiency-inhibited diabetic atherosclerotic calcification. Notably, among the 3 BCKA, both KMV and KIV play a more important role rather than KIC in the regulation of diabetic arterial calcification. Furthermore, prop-CoA is the product of KMV and KIV catabolism and a source of the propionyl group to participate in protein propionylation to regulate gene expression [18,19]. Epigenetic modification related to the level of metabolite is essential for VC [16,38–41]. NR4A3-mediated lactate generation promotes histone lactylation, which contributes to the activation of Phospho1 expression, thereby accelerating arterial calcification [42]. Our results revealed that the level of histone propionylation levels was increased in calcification of diabetic plaque, while VSMC-specific BCTA2 deficiency reduced these levels. H3K23pr is the most differentially affected histone propionylation modification in response to VSMC-specific BCTA2 deficiency, and BCKA supplementation reversed this effect. RUNX2, a member of runt-related transcription factor family, and is essential for osteoblast differentiation of VSMCs and the development of atherosclerotic calcification [43–45]. Furthermore, we observed that H3K23pr enrichment was on the promoter of RUNX2 in response to the BCAT2–BCKA axis. The results are consistent with those of previous studies in which H3K23pr was reported to be highly up-regulated in multiple cardiovascular diseases and enriched on gene promoters to regulate gene expression [25,46–48]. P300 is the most extensively studied modifying enzyme involved in regulating histone propionylation in previous studies. In in vitro experiments, P300 can transfer prop-CoA to lysine sites of histone H3 and H4 (such as H3K23 and H4K8) [49–52]. Consistently, we found that the inhibition of P300 reduced H3K23pr levels, RUNX2 expression, osteoblastic differentiation, and calcium nodule formation.

BAY-069 is an efficient BCAT1/2 dual inhibitor with good cellular activity and selectivity [53]. In this study, BAY-069 effectively inhibited the expression of BCAT2 and its enzyme activity and reduced the osteogenic differentiation of VSMCs and the formation of calcification in diabetic atherosclerotic plaques. Indeed, the BAY-069 used in this study has inhibitory effects on both BCAT1 and BCAT2, and further exploration focusing solely on BCAT2 inhibitors will be another research focus in the future. At present, a study has discovered for the first time a series of potent BCAT2 inhibitors based on the template of 2-benzylaminopyrazolo[1,5-a] pyrimidinone-3-carbonitrile, using a complementary strategy of fragment screening and high-throughput screening (HTS) [54]. However, this inhibitor cannot be obtained. Overall, the inhibitors specifically targeting BCAT2 in the treatment of calcification in diabetic atherosclerotic plaque still need to be further explored. At present, the specific mechanism of diabetic atherosclerotic calcification has not been fully clarified, and there is still a lack of effective intervention to prevent or treat VC in the clinic. Therefore, this study firstly found that targeting BCAT2 may be an effective method to treat diabetic atherosclerotic calcification, which is of great significance.

It is important to acknowledge the limitations of this study. The BAY-069 used in this study has deficiencies in specifically targeting BCAT2 for the treatment of calcification in diabetic plaques. The use of new technical means to achieve specific targeting of BCAT2 inhibitors in VSMCs needs further exploration, and more clinical research is warranted to confirm the benefit observed.

**Fig. 9.**
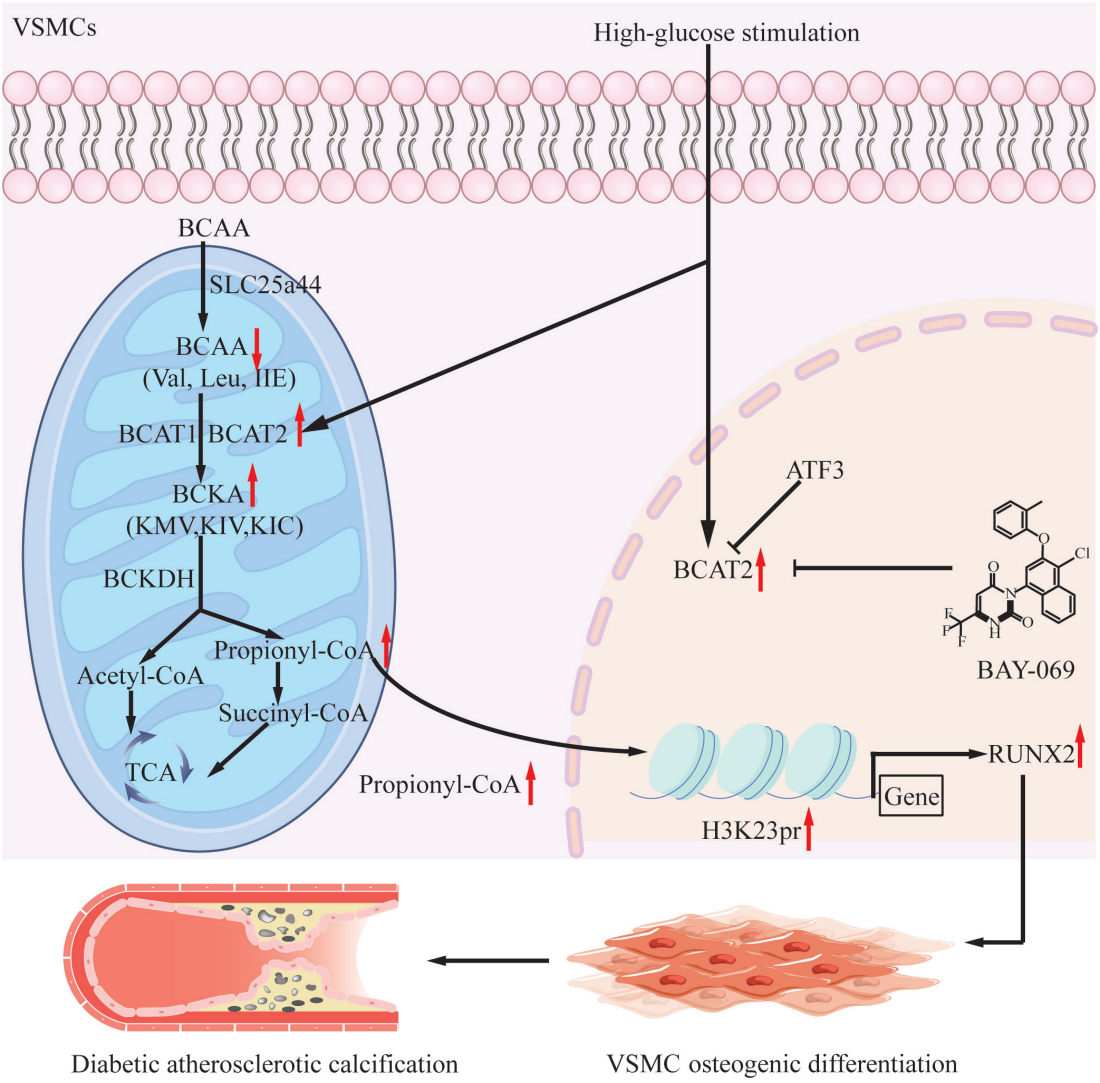
Schematic illustration of BCAT2-mediated BCAA catabolism contributing to the pathogenesis of diabetic atherosclerotic calcification. High glucose stimulates up-regulation of BCAT2 expression and enhances BCAA catabolism. The up-regulation of propionyl-CoA derived from BCKA promotes histone propionylation, thus activating the expression of RUNX2, thereby promoting VSMC osteogenic differentiation and diabetic atherosclerotic plaques.

Our study delineates a previously unrecognized mechanism linking BCAT2-mediated BCAA catabolism with diabetic atherosclerotic calcification. BCAT2-mediated histone propionylation up-regulated RUNX2 expression, resulting in VSMC osteoblastic differentiation and the development of VC in diabetic plaques. Therefore, a specific and effective inhibitor of BCAT2 may be a potential treatment to prevent diabetic arterial calcification (Fig. 9F).

## Materials and Methods

### Reagents

Dulbecco’s modified Eagle’s medium (DMEM), fetal bovine serum (FBS), trypsin, and penicillin/streptomycin were obtained from Wisent (Nanjing, China). H&E staining kit (catalog no. G1121), ascorbic acid (catalog no. A8100), and neutral red staining solution (catalog no. N8160) were purchased from Solarbio (Beijing, China). Von Kossa staining kit (catalog no. R22064), alizarin red S staining solution (catalog no. R23121), and oxidized low-density lipoprotein (ox-LDL; catalog no. YB-002) were from Yuanye (Shanghai, China). β-Glycerophosphate, 2-(4-amidinophenyl)-6-indolecarbamidine dihydrochloride (DAPI; catalog no. D9542), valine (catalog no. 72184), leucine (catalog no. 61905), isoleucine (catalog no. 73325), and prop-CoA (catalog no. 108347-84-8) were from Sigma-Aldrich (USA). KMV (catalog no. M813122), KIV (catalog no. M838960), and KIC (catalog no. M863189) were from Macklin (Shanghai, China). BAY-069 (catalog no. HY-148242) and C646 (catalog no. HY-13823) were obtained from MedChemExpress (New Jersey, USA). Lipo8000 Transfection reagent (catalog no. C0533), calcium content detection kits (catalog no. P0010), bicinchoninic acid (BCA) protein detection kits (catalog no. S1063S), and ALP detection kits (catalog no. P0321M) were from Beyotime (Shanghai, China). Branched-chain amino acid transaminase (BCAT; catalog no. ADS-W-N026) activity assay kit was from Aidisheng (Jiangsu, China). The chromatin immunoprecipitation kit (catalog no. 56383) was from Cell Signaling Technology (Boston, USA).

### Patient sample collections

Human anterior tibial arteries used in this study were collected from individuals who underwent diabetic foot amputation in the Affiliated Hospital of Jiangsu University. Inclusion criteria were as follows: (a) patients aged 18 to 90 years old; (b) patients who were diagnosed with type 2 diabetes; (c) patients who had diabetic foot and had indications for amputation; and (d) complete relevant clinical data. Exclusion criteria were as follows: (a) patients with chronic kidney disease or other diseases that significantly affect VC; (b) patients with severe hepatic and renal insufficiency and other organ dysfunction; (c) patients with malignant tumor; and (d) vulnerable groups such as mental cognitive–behavioral disorders. According to the von Kossa staining of all anterior tibial artery sections, the samples were divided into the NVC group (*n* = 13) and the VC group (*n* = 13). All samples were only used for research objectives, and the informed consent forms have been signed. All protocols and procedures including human samples were approved by the Ethics Committee of the Affiliated Hospital of Jiangsu University (approval ID: KY2021K1224) and were carried out in line with the principles outlined by the Declaration of Helsinki.

### Animals

Tagln is currently a commonly used cre tool mouse promoter for conditional knockout of VSMCs [20]. ApoE^−/−^and Tagln-iCre mice in C57BL/6 background were purchased from GemPharmatech. BCAT2^fl/fl^/ApoE^−/−^ mice were generated by GemPharmatech (China). Exons 4 to 6 of the BCAT2 gene in the ApoE^−/−^mice background were flanked with loxP sites for conditional gene targeting. ApoE^−/−^/Tagln-iCre were generated by crossing ApoE^−/−^ mice with Tagln-iCre, and then crossed into BCAT2^fl/fl^/ApoE^−/−^ mice to form ApoE^−/−^/BCAT2^f/f^/Tagln-iCre (ApoE^−/−/^BCAT2^ΔSMC^) mice.

For diabetic atherosclerotic calcification model, 6-week-old male or female mice were injected with streptozocin (40 mg/kg/day) intraperitoneally for 5 consecutive days. Two weeks after the injection, the fasting blood glucose of mice was measured, and mice with a measurement value of ≥16.7 mM were involved in the experiment as a DM group. Then, these mice were fed with HFD (41 kcal% fat and 43 kcal% carbohydrates) (HFK Bioscience, Beijing, China) for 24 weeks. At the same time, the NC group was injected with an equal volume of citrate buffer as a vehicle control for 5 d and fed with HFD for 24 weeks.

For lentivirus (LV) injection, mice were placed in an injection holder. After wiping and disinfecting with alcohol, the syringe drew 100 μl of diluted LV (10^10^ viral genomes/mouse, pengersi, China) and slowly injected into the tail vein of mice.

For BCKA supplementation in vivo, ApoE^−/−^/BCAT2^ΔSMC^ mice were supplemented with 1 mg/ml BCKA (Sigma, USA) in drinking water along with continued consumption of HFD for 24 weeks. BCKA was first dissolved in dimethyl sulfoxide (DMSO) and then diluted further in drinking water.

For BAY-069 supplementation in vivo, after a 16-week HFD challenge, ApoE^−/−^ mice were injected with BAY-069 (0.3 mg/kg) or DMSO via the caudal vein route every 48 h for 8 weeks, along with continued consumption of HFD. BAY-069 was first dissolved in DMSO and then diluted further in 0.9% physiological saline.

All animal experiments in this study were conducted in accordance with the Guide for the Care and Use of Laboratory Animals published by the National Academy of Sciences and the National Institutes of Health and approved by the Experimental Animal Use Ethics Committee of Jiangsu University (approval ID: SYXK2023-0081). All mice were raised at the Experimental Animal Center of Jiangsu University in specific pathogen-free (SPF) conditions. Euthanasia of the animals was performed in a carbon dioxide chamber. Blood was collected, and aortic roots and whole aortas were carefully dissected for further analyses. Serum levels of total cholesterol and triglyceride were measured using specific reagents.

### Cell culture

Primary VSMCs were isolated from mouse aorta as described previously [2] and cultured in DMEM/F12 with 1.0 g/l glucose, 20% FBS, and 1% penicillin/streptomycin. Primary VSMCs were used for subsequent experiments at passages 3 to 5. Mouse aortic smooth muscle cells (Movas) were purchased from the American Type Culture Collection (ATCC) (Grand Island, NY) and cultured in DMEM with 1.0 g/l glucose, 10% FBS, and 1% penicillin/streptomycin. To establish an in vitro model of diabetic atherosclerotic calcification, cells were cultured in osteogenic medium (OM) for 14 d as OM groups. The OM was composed of high-glucose DMEM (4.5 g/l glucose) supplemented with 10% fetal bovine serum (FBS), 50 μg/ml oxidized low-density lipoprotein (ox-LDL), 1% penicillin/streptomycin, 2.5 mM β-glycerophosphate, and 50 μg/ml ascorbic acid. At the same time, the NM group was cultured in DMEM with 4.5 g/l glucose, 10% FBS, 50 μg/ml ox-LDL, and 1% penicillin/streptomycin for 14 d.

### Spatial metabolome analysis

AFADESI-MSI was employed to investigate the spatial distribution of metabolites in frozen arterial sections obtained from diabetic foot amputations. The raw MS data were converted into the imzML file format using the imzMLConverter software and subsequently processed in the Cardinal software package. This processing included steps for background subtraction, peak alignment, and peak filtering. High-resolution MS imaging data were annotated using the SmetDB database in conjunction with the pySM annotation framework. Differential metabolites between the NVC and VC groups were identified based on a variable importance in projection (VIP) score >1 from the OPLS-DA model and a significance threshold of *P* < 0.05 from a *t* test. Finally, pathway enrichment analysis of the identified differential metabolites was conducted using the KEGG database to elucidate the involvement of metabolic pathways.

### LC-MS/MS assay

Cells were lysed using an ultrapure water extract containing protease inhibitors, phenylmethylsulfonyl fluoride (PMSF), and EDTA, followed by the addition of methanol. The lysates were centrifuged, and the resulting supernatant was collected for analysis. Two-microliter aliquot of the supernatant was injected into an ultra-performance liquid chromatography (UPLC) system (ExionLC AD), which was coupled online to an MS/MS platform (QTRAP 6500+). The electrospray ionization (ESI) source was maintained at 550 °C, with a mass spectrometer voltage of 5,500 V in positive ion mode and −4,500 V in negative ion mode. The curtain gas (CUR) pressure was set to 35 psi. In the QTRAP 6500+ system, ion pairs were scanned and detected using optimized declustering potential (DP) and collision energy (CE) for each metabolite. Metabolite levels were normalized to protein concentration to account for variations in sample input.

### ScRNA-seq analysis

ScRNA-seq datasets from arteries of diabetic foot amputations were used (GSE248609). The samples were analyzed to the NVC and VC group by the lower extremity CT. Seurat (https://satijalab.org/seurat/) package was used for cell normalization and cell filtering considering the mitochondrion percentage and minimum and maximum gene numbers. Principal components analysis (PCA) and *t*-distributed stochastic neighbor embedding (t-SNE) analysis was used for the single cell to cell relation description. Graphcluster and *K*-mean were utilized for cell clustering, and Wilcox rank sum test was used for marker gene analysis. SingleR and CellMarker databases were used to annotate cell types. Uniform Manifold Approximation and Projection (UMAP) analysis was used to represent cell cluster. The SCENIC analysis was run as described on the cells that passed the filtering, using the 20,000 motifs database for RcisTarget and GRNboost. The R package QuSAGE was used for gene set enrichment analysis to achieve the enrichment status and enrich significance of each gene sets.

### RNA-seq assay

Total RNA was extracted from aorta tissues using Trizol. RNA sample quality was assessed by Qubit 4.0 fluorescence meter and Qsep400 Bioanalyzer. Then, a cDNA library was constructed and the quality of the library was tested by the Qubit dye method and qPCR. Illumina platform was used to sequence. Raw data (raw reads) of FASTQ format were firstly processed through in-house perl scripts. Clean data (clean reads) were obtained by removing the raw reads that contained adapters, reads containing ploy-*N*, and low-quality reads from raw data. For positional information on the reference genome or gene, as well as unique sequence feature information of the sequencing sample, sequence alignment between clean reads and the reference genome was performed by HISAT2. Differential expression analysis of 2 groups was performed by DESeq2. The screening criteria for differential expression genes are log_2_Fold Change ≥ 1 and false discovery rate (FDR) < 0.05.

### CoA quantification

Cells were mixed with 600 μl of methanol, vortexed thoroughly, and transferred to 2-ml grinding tubes. After grinding at 60 Hz, 600 μl of chloroform was added, followed by shaking; subsequently, 240 μl of ultrapure water was added, and the mixture was incubated on ice for 10 min. The sample was centrifuged at 4 °C and 12,000 rpm for 2 min, and 250 μl of the supernatant was taken into a new Eppendorf (EP) tube and dried in a vacuum centrifuge concentrator. The sample was dissolved in 50 μl of methanol/water solution (3:2, v/v) and centrifuged, and 35 μl of the resulting supernatant was collected for subsequent LC-MS/MS analysis. Metabolites were normalized on cell number.

### BCAT activity assay

 Samples were mixed with the extraction solution and subjected to ultrasonic crushing. The supernatant was taken by centrifugation for detection. The experimental steps were performed followed by BCAT activity assay kit. Absorbance at 450 nm was measured to calculate the activity of BCAT. Cell numbers were counted for normalization.

### In vivo calcification evaluation

The whole aorta of calcification was identified by alizarin red S staining. Briefly, samples were fixed in 95% ethanol for 24 h and then stained with 0.003% alizarin red S solution in 1% potassium hydroxide overnight. The aorta was rinsed in 2% potassium hydroxide and photographed.

Tissue sections of calcification were identified using von Kossa stain kit and analyzed using ImageJ. Briefly, paraffin sections were deparaffinized, hydrated, and performed according to the instructions. Finally, samples were stained with 1% neutral red for 3 min and observed under a microscope (IX51, Olympus, Japan). Calcified spots within the medial aortic layer were evaluated based on percentage of calcification lesion size (% of plaque area).

Calcium content determination of aorta was quantified using the Calcium Assay Kit. Total protein concentration was detected by BCA protein assay. Calcium content was calibrated by the protein concentration. ALP activity in aortas was quantified using the ALP assay kit. ALP activity was calibrated by the weight of aorta. All experimental steps were in compliance with the manufacturer’s protocol.

### Micro-CT detection

The whole aorta of calcification was identified by micro-CT. Briefly, samples were fixed in 4% paraformaldehyde for 24 h and then detected with the micro-CT scanning device (NMC-200 Nemo, PINGSENG Healthcare, Suzhou, China) at a resolution of 10 μm.

### In vitro calcification evaluation

Cells were fixed in 4% paraformaldehyde for 30 min and then stained in 2% alizarin red S solution (pH 4.2) at room temperature for 15 min. Finally, cells were observed and photographed. Calcium content of cells was analyzed as described above.

### Immunohistochemistry and immunofluorescence staining

For immunohistochemistry staining, paraffin sections were deparaffinized and hydrated followed by boiling for antigen retrieval. The experimental steps were performed following the SP Rabbit & Mouse HRP kit (CW2069S, CWBIO, China). Images were recorded using a microscope (IX51, Olympus, Japan). ImageJ software was used for image analyses. Five different visual fields in one specimen were randomly selected for quantification of positive staining cells. The primary antibodies used were anti-RUNX2 (ab236639, Abcam, USA).

For immunofluorescence staining, cryosections or cells were washed with phosphate-buffered saline (PBS) and fixed in 4% paraformaldehyde for 30 min. Samples were incubated with 5% serum and 0.3% Triton X-100 for 1 h to block nonspecific binding. Samples were incubated with primary antibodies, including anti-BCAT2 (Ab309514, Abcam, USA), anti-Kpr (PTM-203, Proteomics, China), anti-H3K23pr (PTM-208, Proteomics, China), anti-ATF3 (sc-518032, Santa Cruz, USA), or anti-α-SMA (BM0002, Boster, China) at 4 °C overnight followed by incubation with corresponding secondary antibody. The secondary antibodies used were goat anti-rabbit IgG (AF488, catalog no. ab0141, Abways, China), goat anti-mouse IgG (AF488, catalog no. ab0142, Abways, China), goat anti-rabbit IgG (AF594, catalog no. ab0151, Abways, China), and goat anti-mouse IgG (AF594, catalog no. ab0152, Abways, China). Nuclei were stained using DAPI. All immunofluorescence images were captured on a microscope (IX51, Olympus, Japan) and quantified using ImageJ (National Institutes of Health, USA). Results were expressed as the ratio of relative florescence intensity compared with the controls.

### qRT-PCR and Western blot

For qRT-PCR, total RNA was extracted from samples by Trizol and reverse transcribed into cDNA with the Reverse Transcriptase Kit, followed by amplification by qPCR with gene-specific primers and SYBR qPCR Master Mix. All experimental steps were performed with the manufacturer’s instructions. The relative mRNA level was calculated by the ∆∆*C*_t_ method using glyceraldehyde-3-phosphate dehydrogenase (GAPDH) as control.

For Western blot, the protein of VSMCs or aortic tissue was extracted by protein lysate and loading buffer, separated on the sodium dodecyl sulfate–polyacrylamide gel electrophoresis (SDS-PAGE) gel, and transferred to a polyvinylidene difluoride (PVDF) membrane. Membranes were incubated in 5% skimmed milk at 37 °C for 1 h followed by incubation with diluted primary antibody at 4 °C overnight. Membranes were then incubated with the secondary antibody for 1 h at 37 °C and developed using a chemiluminescence system (Amersham Imager 600, General Electric, Boston, USA). ImageJ software was used to quantify band intensities. The primary antibodies used were anti-BCAT1 (13640-1-AP, Proteintech, China), anti-BCAT2 (16417-1-AP, Proteintech, China), anti-RUNX2 (ab236639, Abcam, USA), anti-BMP2 (66383-1-lg, Proteintech, China), anti-α-SMA (BM0002, Boster, China), anti-sm22α (a6760, ABclonal, China), anti-β-actin (Ab8226, Abcam, USA), anti-kpr (PTM-203, Proteomics, China), anti-H3K14pr (PTM-211, Proteomics, China), anti-H3K28pr (PTM-214, Proteomics, China), anti-H3K23pr (PTM-208, Proteomics, China), anti-H3K56pr (PTM-220, Proteomics, China), anti-histone H3 (CY6587, Abways, China), and anti-P300 (20695-1-AP, Proteintech, China). The secondary antibodies used were goat anti-rabbit IgG (HRP, catalog no. ab0101, Abways, China) and goat anti-mouse IgG (horseradish peroxidase, catalog no. ab0102, Abways, China).

### LV transfection

Movas were infected with recombinant LVs expressing scrambled short hairpin RNA (shRNA) or BCAT2 shRNA mixed with serum-free medium and polybrene. Twelve hours after infection, the medium was changed to normal growth medium. After 72 h, cells were added with 2 μg/ml puromycin and incubated at 37 °C and 5% CO_2_ for 24 h to screen for stable transformants.

### Plasmid or siRNA transfection

Cells were transfected with plasmids or siRNA mixed with Opti-MEM medium and Lipo8000 reagent (Beyotime, China) when the cell density reached 70% to 80% confluence. Experimental steps were in accordance with the manufacturer’s protocol of Lipo8000 reagent. The protein expression in cells transfected with plasmids or siRNA was examined by Western blot analysis.

### ChIP assay

ChIP was performed using a Chromatin IP Kit (Cell Signaling Technology, Massachusetts, USA) following the manufacturer’s protocol. Briefly, cells were cross-linked with 37% formaldehyde for 10 min, and the reaction was quenched by the addition of glycine. Nuclei were extracted, and chromatin was fragmented to an average size of 200 to 800 bp via sonication. Chromatin samples were incubated overnight at 4 °C with either ATF3 or H3K23pr primary antibodies, or normal IgG as a control, under constant rotation. Protein G magnetic beads were then added and incubated for 2 h at 4 °C. Following magnetic separation, the supernatant was discarded, and the beads were washed and eluted. NaCl and proteinase K were added, and cross-links were reversed by incubation at 65 °C overnight. The DNA was purified using a spin column and analyzed by qRT-PCR to detect antibody-bound DNA fragments.

The qPCR primers used to evaluate the promoter regions were as follows: BCAT2, GCACCGATTGTCTAACTACCTCTG (forward) and TCAAATCATTACAGGACCTCACATCAG (reverse); RUNX2, CACCATCACAGTCATCCGTTCC (forward) and AAAGGCGAGCAGACCAATTTCC (reverse).

### Molecular docking

Protein Data Bank (BCAT2, PDB: 5mpr) and PubChem (BAY-069, PubChem CID: 155555842) were used to download the molecular structure. Docking was processed by Auto Dock software and visualized through Pymol software.

### Histological staining

For H&E staining, paraffin sections were deparaffinized and hydrated. The experimental steps were performed followed by the H&E staining kit. All images were captured on a microscope and quantified using ImageJ (National Institutes of Health, USA).

For oil red O staining, cryosections were washed with PBS and fixed in 4% paraformaldehyde for 30 min. Oil red O solution was diluted in double-distilled water (ddH_2_O) at 3:2 and filtered to make a working solution. The sections were incubated with isopropanol diluted in ddH_2_O at 3:2 for 15 s and then stained with oil red O working solution for 30 min. All images were captured on a microscope (Olympus, Japan) and quantified using ImageJ (National Institutes of Health, USA).

### Statistical analysis

Statistical analysis was performed using SPSS version 25.0 or GraphPad Prism 9.0. Values are expressed as mean ± SD for continuous variables or *n* (%) for categorical variables. Categorical variables were assessed using chi-square test. Data normality was assessed by Shapiro–Wilk test, and homogeneity of variance was assessed by *F* test. Single comparisons between 2 groups were performed by unpaired, 2-tailed Student’s *t* test. Multiple comparisons were performed by 1-way analysis of variance (ANOVA) with Dunnett’s post hoc test or 2-way ANOVA with Tukey’s post hoc test. *P < 0.05, **P < 0.01, ***P < 0.001, ****P < 0.0001 were considered statistically significant.

## Data Availability

The datasets during the current study are available from the corresponding author on reasonable request.
