# Supplementary material for: Vascular Smooth Muscle Cell-Specific BCAT2 Deficiency Attenuates Diabetic Atherosclerotic Calcification via Histone Propionylation
Source: Research (Wash D C). 2026 Jan 6;9:1052. doi: 10.34133/research.1052 (PMC12770235; doi:10.34133/research.1052)
Supplement: Supplementary 1 — Figs. S1 to S15 Table S1 [file research.1052.f1.docx]

**Supplementary figures**


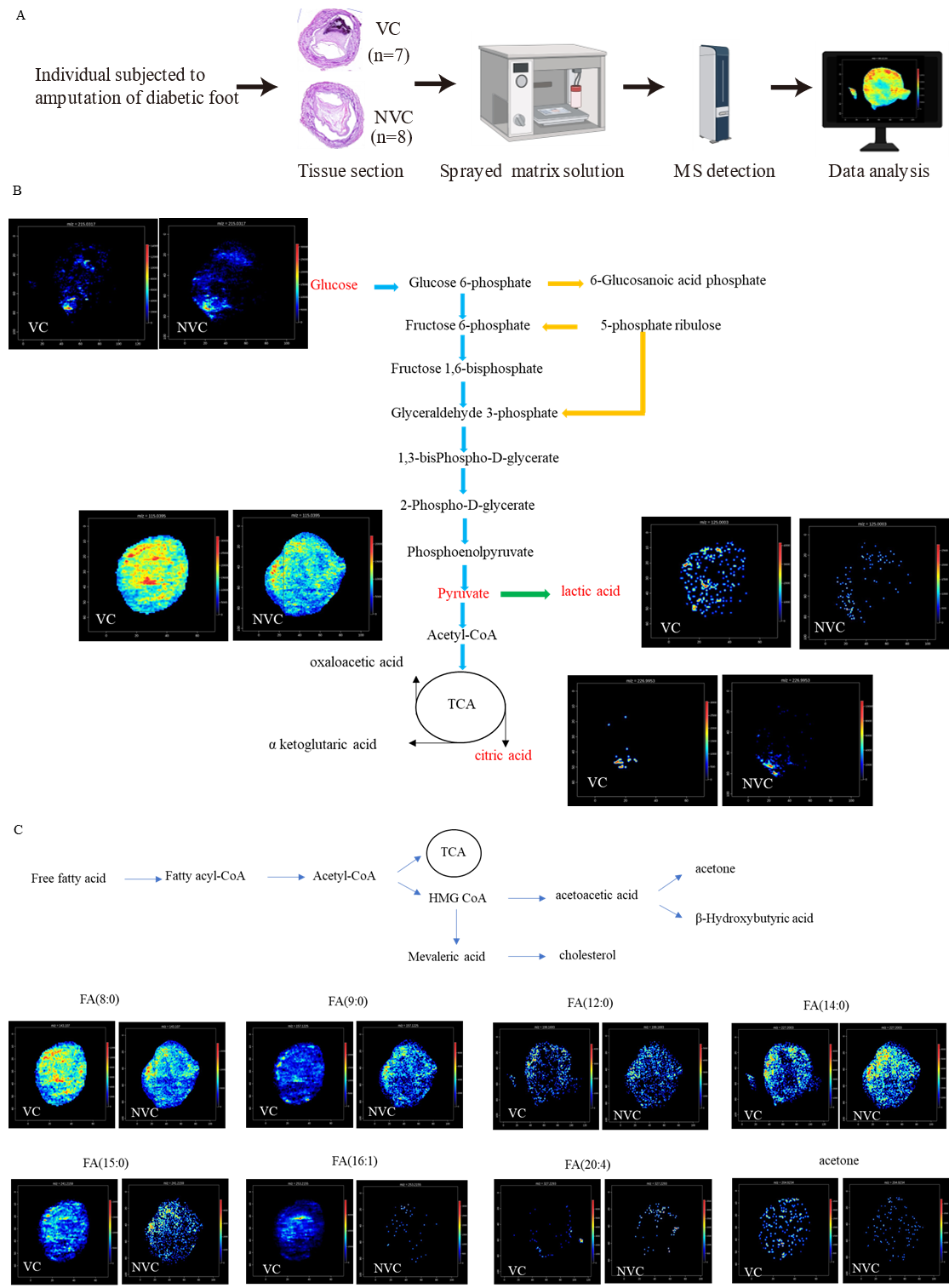


**Figure S1.** Spatial metabonomic analysis of anterior tibial artery in patients with diabetic foot amputation. (A) Schematic showing the spatial metabolomics-based experimental design using anterior tibial artery samples collected from individuals subjected to diabetic foot amputation. The element images are sourced from www.BioRender.com. (B) Schematic diagram of glucose metabolism pathway, as well as mass spectra of differential metabolites in VC and NVC groups, including Glucose, pyruvate, lactic acid, citric acid. (C) Schematic diagram of lipid metabolism pathway and mass spectrometry of differential metabolites in VC and NVC groups.


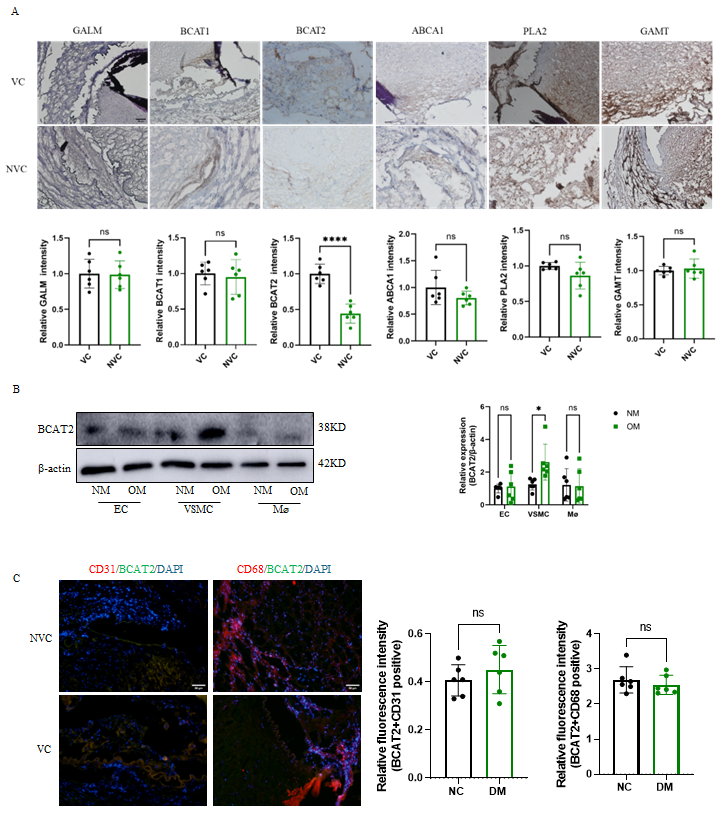


**Figure S2.** BCAT2 was upregulated in VSMCs. (A) Representative images and quantification of immunohistochemistry staining of GALM, BCAT1, BCAT2, ABCA1, PLA2 and GAMT in anterior tibial artery sections. Scale bars, 50μm. n=6. **** *P* < 0.0001*vs.* VC. (B) Western blot image and analysis showing BCAT2 protein expression level in EC, VSMC or Mø treated with NM or OM for 14 days. n=6. **P* < 0.05 *vs.* NM. (C) Representative images immunofluorescence staining of BCAT2 and α-SMA in anterior tibial artery sections. Red: CD31/CD68, Green: BCAT2, Blue: DAPI. scale bar, 50μm. n=6. Data are presented as mean±SD. ns, not significant.


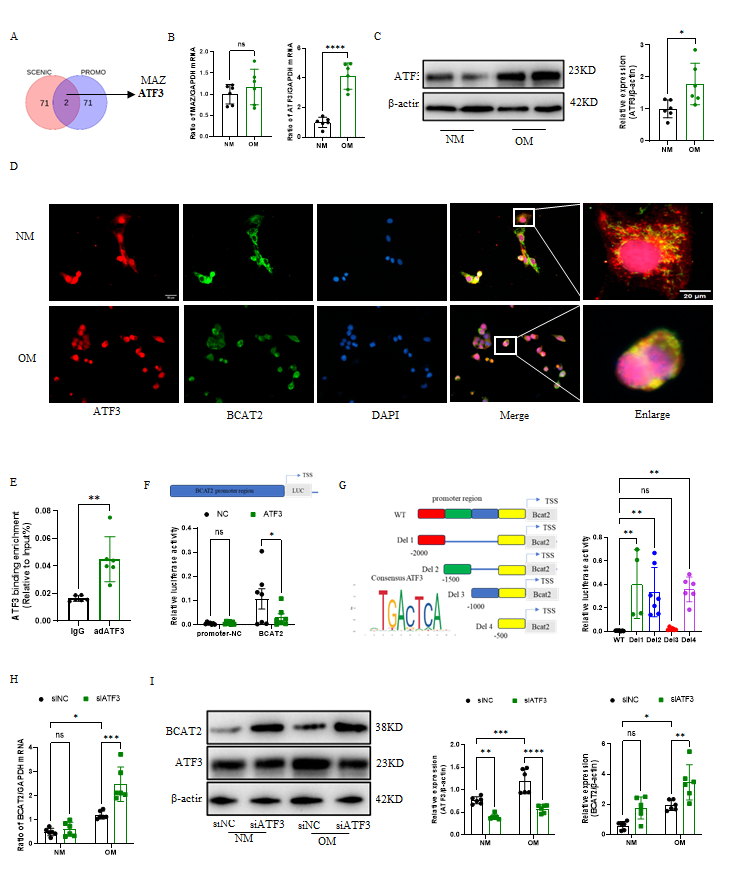


**Figure S3.** ATF3 negatively regulates BCAT2 expression. (A) SCESIC analyses of scRNA-sequencing data, accompanied by transcription factors predicted by PROMO to identify potential upstream targets of BCAT2. (B) qRT-PCR analysis of ATF3 and MAZ gene expression in Movas treated with NM or OM for 14 days. n=6. **** *P* < 0.0001*vs.* NM. (C) Western blot image and analysis showing ATF3 protein expression level in movas treated with NM or OM for 14 days. n=6. **P* < 0.05 *vs.* NM. (D) Representative images of immunofluorescence staining of BCAT2 and ATF3 in Movas treated with NM or OM. n=6. Red: ATF3, Green: BCAT2, Blue: DAPI. scale bar, 50μm. (E) ChIP assay was used to detect the binding of ATF3 to BCAT2 promoter. n=6. ***P* < 0.001 *vs.* IgG. (F) Luciferase reporter gene assays were used to detect the binding of ATF3 to BCAT2 promoter. n=6. **P* < 0.05 *vs.* NC. (G) Luciferase reporter gene assays were used to detect the binding of ATF3 to BCAT2 promoter and its truncated forms. ***P* < 0.001 *vs.* WT. (H) qRT-PCR analysis of BCAT2 gene expression in Movas transfected with negative control siRNA (siNC) or ATF3 siRNA (siATF3) under NM or OM treatment for 14 days. n=6. * *P* < 0.05, *** *P* < 0.001 *vs.* siNC. (I) Western blot image and analysis showing ATF3 and BCAT2 protein expression level in movas transfected with siNC or siATF3 under NM or OM treatment for 14 days. n=6. * *P* < 0.05, ** *P* < 0.01, *** *P* < 0.001, **** *P* < 0.0001 *vs.* siNC. Data are presented as mean±SD. ns, not significant.

**Figure S4.** Knockdown of BCAT2 inhibits calcification in diabetic plaque. (A) Schematic protocol of diabetic ApoE^-/-^mice transfected with LV-shNC or LV-shBCAT2. (B) and (C) Biochemical measurement of aortic calcium content and ALP activity. n=6. (D) and (E) Alizarin red S staining and Micro-CT of the whole mouse aortic calcification. (F) Western blot image and analysis showing SM22α, α-SMA, BMP2, RUNX2 and BCAT2 protein expression level. n=6. (G) Representative images and quantification of HE and von Kossa staining in aortic arch and aortic root tissue sections. Scale bars, 200μm. n=6. Data are presented as mean±SD.**P* < 0.05, ***P* < 0.01, *****P* < 0.0001 *vs.* LV-shNC.

**Figure S5.** Establishment of ApoE^-/-^/BCAT2^ΔSMC^ mice. (A) Schematic representation of the construction of VSMC-specific BCAT2 knockout mice. (B) Agarose gel electrophoresis imaging of PCR amplification products of genomic DNA. Lane 1-19 represents the offspring of ApoE^-/-^ BCAT2^fl/fl^ mice and ApoE^-/-^ Tagln^Cre^ hybrid mice, while Lane B6 represents C57BL/6J wild-type mice, serving as a negative control (C) Representative images and quantification of immunofluorescence staining of BCAT2 and α-SMA in aortic sections of ApoE^-/-^ BCAT2^fl/fl^ and ApoE^-/-^/BCAT2^ΔSMC^ mice. Red: BCAT2， Green: α-SMA， Blue: DAPI， Scale bar, 50 μm. (C) Representative images and quantification of immunofluorescence staining of BCAT2 and α-SMA in VSMCs from ApoE^-/-^/BCAT2^fl/fl^ and ApoE^-/-^/BCAT2^ΔSMC^ mice. Red: BCAT2, Green: α-SMA, Blue: DAPI. scale bar, 50μm. n=6. Data are presented as mean±SD.*****P* < 0.0001 *vs.* ApoE^-/-^/BCAT2^fl/fl^.

**Figure S6.** VMSCs-specific BCAT2 deficiency inhibits diabetic atherosclerotic calcification without gender bias. (A) Western blot image and analysis showing SM22α, α-SMA, BMP2 and RUNX2 protein expression level in the aorta of ApoE^-/-^/BCAT2^fl/fl^ and ApoE^-/-^/BCAT2^ΔSMC^ mice. n = 6. (B) and (C) Biochemical measurement of aortic calcium content and ALP activity for indicated mice. n=6. (D) Representative images and quantification of HE and von Kossa staining in aortic arch and aortic root tissue sections. Scale bars, 200μm. n=6. Data are presented as mean±SD.**P* < 0.05, ***P* < 0.01, *****P* < 0.0001 *vs.* ApoE^-/-^/BCAT2^fl/fl^.

**Figure S7.** Knockdown of BCAT2 inhibits calcification in Movas. (A) Western blot image and analysis showing SM22α, α-SMA, BMP2, RUNX2 and BCAT2 protein expression level in Movas transfected with LV-shNC or LV-shBCAT2 under NM or OM treatment for 14 days. n=6. (B) Representative images of alizarin red S staining in Movas transfected with LV-shNC or LV-shBCAT2 under NM or OM treatment for 14 days. Scale bars, 200μm. n=6. (C) Quantification of calcium deposits. n=6. Data are presented as mean±SD.**P* < 0.05, ***P* < 0.01, ****P* < 0.001, *****P* < 0.0001 *vs.* LV-shNC. ns, not significant.

**Figure S8.** Overexpression of BCAT2 promotes calcification in Movas. (A) Western blot image and analysis showing SM22α, α-SMA, BMP2, RUNX2 and BCAT2 protein expression level in movas transfected with adcon or adBCAT2 under NM or OM treatment for 14 days. n=6. (B) Representative images of alizarin red S staining in movas transfected with adcon or adBCAT2 under NM or OM treatment for 14 days. Scale bars, 200μm. n=6. (C) Quantification of calcium deposits. n=6. Data are presented as mean±SD.**P* < 0.05, ***P* < 0.01, *****P* < 0.0001 *vs.* adcon. ns, not significant.

**Figure S9.** High BCAA promotes diabetic atherosclerotic calcification. (A) Representative images and quantification of HE and von Kossa staining in aortic arch and aortic root tissue sections. Scale bars, 200μm. n=6. ****P* < 0.001 *vs.* NC. (B) and (C) Biochemical measurement of aortic calcium content and ALP activity. n=6. ****P* < 0.001 *vs.* NC. (D) and (E) Representative images of alizarin red S staining and quantification of calcium deposits in movas treated with 800μM or 1600μM BCAA under OM treatment for 14 days. Scale bars, 200μm. n=6. (F) and (G) Representative images of alizarin red S staining and quantification of calcium deposits in movas treated with 800μM BCAA, 1600μM VAL, 1600μM IIE or 1600μM LEU under NM or OM treatment for 14 days. Scale bars, 200μm. n=6. *****P* < 0.0001 *vs.* 800μM BCAA. Data are presented as mean±SD.


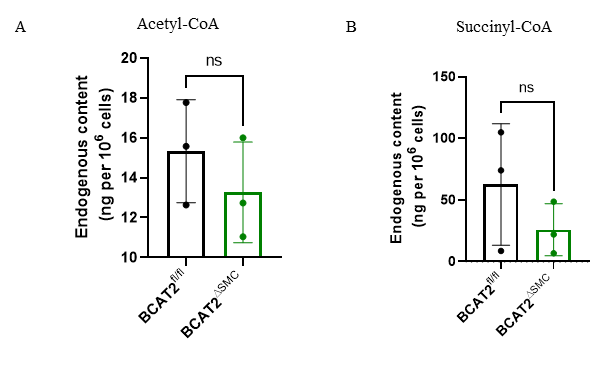


**Figure S10.** The concentration of acetyl-CoA or succinyl-CoA. (A) and (B) Quantification of acetyl-CoA or succinyl-CoA. in VSMCs from ApoE^-/-^/BCAT2^fl/fl^ and ApoE^-/-^/BCAT2^ΔSMC^ mice under OM treatment for 14 days. n = 3. Data are presented as mean±SD. ns, not significant.

**Figure S11.** Knockdown of BCAT2 inhibits histone propionylation in Movas. (A) and (B) Representative images and quantification of of immunofluorescence staining of Kpr or H3K23pr in Movas transfected with LV-shNC or LV-shBCAT2 under OM treatment. Red: Kpr/H3K23pr, Blue: DAPI. scale bar, 50μm. n=6. ****P* < 0.001, *****P* < 0.0001 *vs.* LV-shNC. (C) Western blot image and analysis showing protein propionylation and H3K23pr protein expression level in Movas transfected with LV-shNC or LV-shBCAT2 under OM treatment for 14 days. *****P* < 0.0001 *vs.* LV-shNC. (D) Western blot image and analysis showing H3K23pr protein expression level in VSMCs from ApoE^-/-^/BCAT2^ΔSMC^ mice supplemented with or without Propionyl-CoA under OM treatment for 14 days. n=6. ****P* < 0.001, *****P* < 0.0001 *vs.* ApoE^-/-^/BCAT2^fl/fl^ or ApoE^-/-^/BCAT2^ΔSMC^. Data are presented as mean±SD.

**Figure S12.** Histone propionylation was increased in calcified arteries from diabetic foot amputation. (A) Left panel, The adjacent Von Kossa-stained tissue images were shown. scale bar, 200μm. Right panel, Representative images of immunofluorescence staining of Kpr and α-SMA. Red: Kpr, Green: α-SMA, Blue: DAPI. scale bar, 50μm. n=13. **** *P* < 0.0001*vs.* NVC. (B) Representative images and quantification of of immunofluorescence staining of H3K23pr and α-SMA in the vascular samples of NVC and VC group. Red: H3K23pr, Green: α-SMA, Blue: DAPI. scale bar, 50μm. n=13. **** *P* < 0.0001*vs.* NVC. (C) and (D) Representative images and quantification of of immunofluorescence staining of Kpr or H3K23pr in Movas treated with NM or OM. Red: Kpr/H3K23pr, Blue: DAPI. scale bar, 50μm. n=6. *** *P* < 0.001, **** *P* < 0.0001*vs.* NM. (E) Western blot image and analysis showing protein propionylation and H3K23pr protein expression level. n=6. * *P* < 0.05, ** *P* < 0.01*vs.* NM. Data are presented as mean±SD.

**Figure S13.** Inhibition of P300 reduces H3K23pr levels and BCKA–induced calcium deposition. (A) and (B) Western blot image and analysis showing RUNX2, BMP2, α-SMA, sm22α, H3K23pr, and P300 protein expression level in VSMCs from ApoE^-/-^/BCAT2^ΔSMC^ mice pretreated with DMSO or C646 (P300 inhibitor) for 48 hours followed by vehicle or BCKA supplement for 24 hours under OM treatment for 14 days. n=6. (C) Representative images of alizarin red S staining in VSMCs. Scale bars, 200μm. n=6. (D) Quantification of calcium deposits. n=6. Data are presented as mean±SD. **P* < 0.001, ****P* < 0.001, ****P* < 0.001, *****P* < 0.0001*vs.* Vehicle or BCKA+DMSO.

Figure S14. BAY-069 inhibits osteogenic differentiation. (A-B) Quantification of Western blot in Figure 8D and 8J. Data are presented as mean±SD. **P* < 0.001, ****P* < 0.001, ****P* < 0.001, *****P* < 0.0001*vs.* NC or NM. ns, not significant.

Figure S15. BAY-069 inhibits diabetic atherosclerosis. (A) Representative in situ images of atherosclerotic plaque in the aortic arch. (B) Oil red O staining of the whole aorta. (C) Representative images of HE staining and Oil red O staining in aortic root sections. Scale bars, 200μm for HE staining, 50μm for Oil red O staining. n=6. Data are presented as mean±SD. * *P* < 0.05, **** *P* < 0.0001*vs.* DMSO. ns, not significant.

**Supplementary tables**

**Table S1** Baseline characteristics of diabetes foot amputee patients

| Characteristic | NVC (n=13) | VC (n=13) | *P* value |
| --- | --- | --- | --- |
| Age (years) | 70.5±12.5 | 70.0±7.8 | 0.896 |
| Male sex, no. (%) | 9(69.2) | 8 (61.5) | 0.680 |
| BMI, kg/m^2^ | 24.0±2.3 | 25.3±2.5 | 0.167 |
| SBP, mmHg | 156.8±21.3 | 168.0±21.0 | 0.188 |
| DBP, mmHg | 93.6±16.2 | 92.5±11.0 | 0.991 |
| Current smoker, no. (%) | 4(30.8) | 3(23.1) | 0.658 |
| Alcohol user, no. (%) | 3(23.1) | 4(30.8) | 0.658 |
| FPG, mmol/L | 6.0±1.2 | 6.8±2.0 | 0.226 |
| HbA1c (%) | 6.2±0.8 | 7.4±1.4 | 0.015 |
| Total cholesterol, mmol/L | 4.4±0.9 | 4.2±0.7 | 0.599 |
| Triglycerides, mmol/L | 1.5±0.9 | 1.9±1.0 | 0.311 |
| HDL cholesterol, mmol/L | 1.2±0.3 | 1.2±0.2 | 0.976 |
| LDL cholesterol, mmol/L | 2.4±0.8 | 2.2±0.4 | 0.797 |

Values are expressed as mean±SD for continuous variables confirmed by Shapiro-Wilk test or n (%) for categorical variables, respectively. Statistical significance was assessed using Student's t-test or Chi-square test. VC, vascular calcification; BMI, body mass index; SBP, systolic blood pressure; DBP, diastolic blood pressure; FPG, fasting plasma glucose; HbA1c, Hemoglobin A1c; HDL-C, high-density lipoprotein cholesterol; LDL-C, low-density lipoprotein cholesterol.
